# Supplementary material for: Airway epithelial CD47 plays a critical role in inducing influenza virus-mediated bacterial super-infection
Source: Nat Commun. 2024 Apr 30;15:3666. doi: 10.1038/s41467-024-47963-5 (PMC11063069; doi:10.1038/s41467-024-47963-5)
Supplement: Supplementary file 1 — Supplementary Information [file 41467_2024_47963_MOESM1_ESM.pdf]

Airway epithelial CD47 plays a critical role in inducing influenza virus-mediated bacterial super-infection

Sungmin Moon[1,2], Seunghan Han[1,2], In-Hwan Jang[3], Jaechan Ryu[4], Min-Seok Rha[5], Hyung-Ju Cho[5,6], Sang Sun Yoon[7], Ki Taek Nam[1], Chang-Hoon Kim[5,6], Man-Seong Park[8], Je Kyung Seong[9,10], Won-Jae Lee[3], Joo-Heon Yoon[5,6], Youn Wook Chung[1,6,*], Ji-Hwan Ryu[1,2,*]

[1]Department of Biomedical Sciences, Yonsei University College of Medicine, Seoul 03722, Republic of Korea.

[2]Brain Korea 21 PLUS Project for Medical Science, Yonsei University College of Medicine, Seoul 03722, Republic of Korea.

[3]National Creative Research Initiative Center for Hologenomics and School of Biological Sciences, Seoul National University, Seoul 08826, Republic of Korea.

[4]Microenvironment and Immunity Unit, Institut Pasteur, INSERM U1224, Paris, France.

[5]Department of Otorhinolaryngology, Yonsei University College of Medicine, Seoul 03722, Republic of Korea.

[6]Airway Mucus Institute, Yonsei University College of Medicine, Seoul 03722, Republic of Korea.

[7]Department of Microbiology and Immunology, Yonsei University College of Medicine, Seoul 03722, Republic of Korea.

[8]Department of Microbiology, Institute for Viral Diseases, Vaccine Innovation Center, Korea University College of Medicine, Seoul 02841, Republic of Korea.

[9]Korea Mouse Phenotyping Center, Seoul National University, Seoul 08826, Republic of Korea.

[10]Laboratory of Developmental Biology and Genomics, College of Veterinary Medicine, Seoul National University, Seoul 08826, Republic of Korea.

*Corresponding author. Email: chungyw@yuhs.ac

*Corresponding author. Email: yjh@yuhs.ac

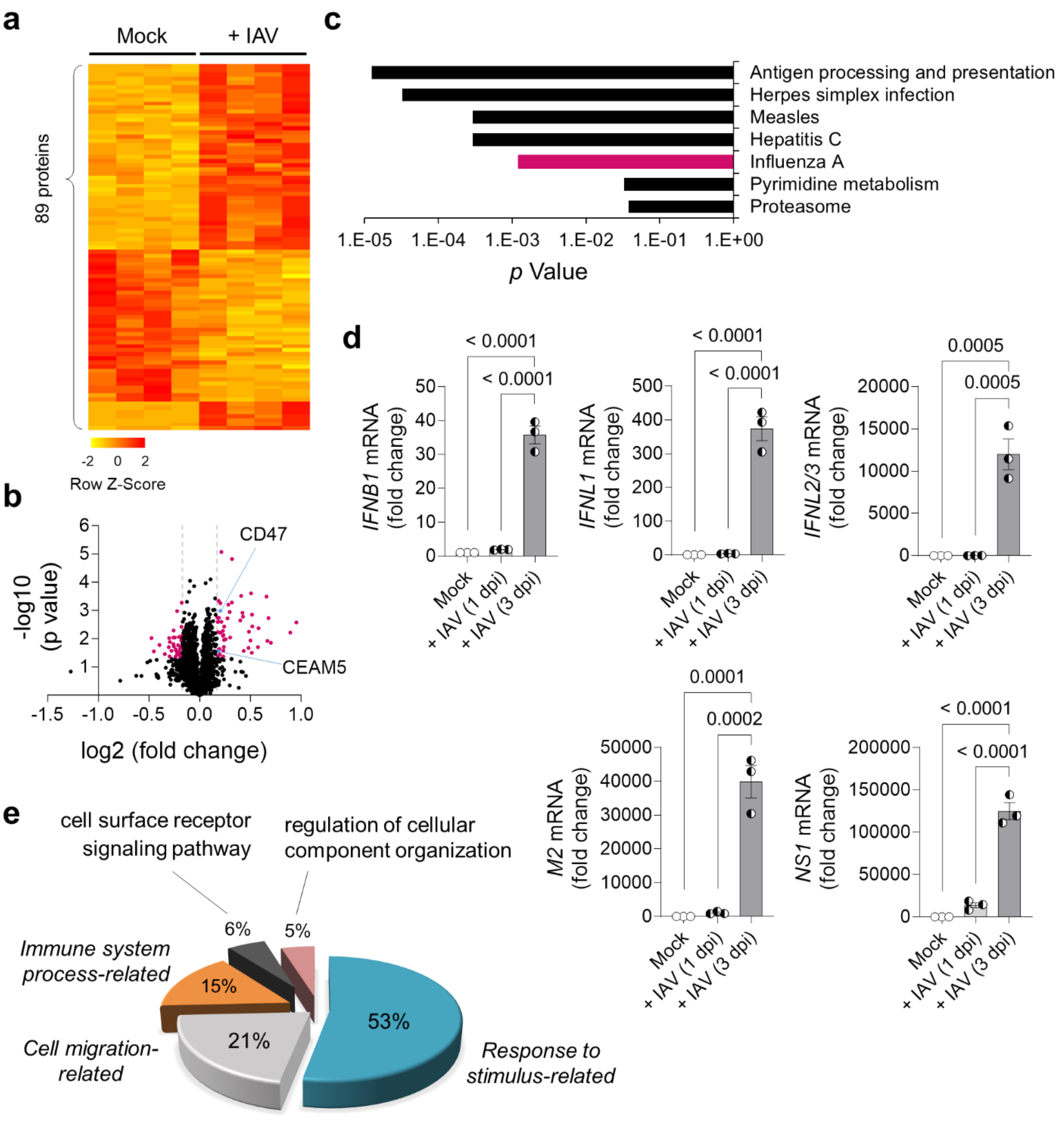

Supplementary Fig. 1. Proteomic analysis of influenza virus-infected airway epithelial cells. Proteomic characterization of human nasal epithelial cells (HNECs), uninfected (*mock*, n = 4) or infected with influenza virus (+ *influenza virus*, n = 4), using Isobaric tags for relative and absolute quantitation (iTRAQ). Cell lysates were harvested on day 5 post-infection (dpi). A total of 3,583 proteins were identified in the iTRAQ-HNECs-Virus dataset. **a** Heat map showing the relative abundance of 89 proteins (FC > 1.125, $p < 0.05$). The color key indicates the relative abundance of each protein (-2 to 2)

across eight samples. **b** Volcano plot demonstrates fold changes in protein abundance between Mock and Virus-only group. The *x*-axis represents the log2 ratio, and the *y*-axis represents significant differences (-log10 of *p*-value). **c** Significant biological pathways enriched among 89 proteins identified via iTRAQ are represented as bar graphs ($p < 0.05$). **d** qPCR analysis of host cell and viral genes at 1 and 3 dpi ($n = 3$). **e** Pie graph showing the CD47-included top 5 biological processes in functional annotation with gene ontology (GO) analysis: *response to stimulus-related* consists of GO:0009605-response to external stimulus, GO:0006952-defense response, GO:0009607-response to biotic stimulus, GO:0043207-response to external biotic stimulus, GO:0051707-response to other organism, GO:0080134-regulation of response to stress, GO:0048584-positive regulation of response to stimulus, GO:0031347-regulation of defense response, GO:0032101-regulation of response to external stimulus, GO:0009617-response to bacterium, and GO:0031349-positive regulation of defense response; *Cell migration-related* consists of GO:0040011-locomotion, GO:0006928-movement of cell or subcellular component, GO:0048870-cell motility, GO:0051674-localization of cell, GO:0016477-cell migration, and GO:0098602-single organism cell adhesion; *Immune system process-related* consists of GO:0002682-regulation of immune system process, GO:0002252-immune effector process, and GO:0002684-positive regulation of immune system process; GO:0007166-cell surface receptor signaling pathway; and GO:0051128-regulation of cellular component organization. Data are presented as mean values ± SEM. Significance was determined by one-way ANOVA with Tukey's multiple comparisons test. *IFNB1*, type I interferon; *IFNL1* and *IFNL2/3*, type III interferons; *M2*, influenza matrix protein 2; *NS1*, influenza non-structural protein 1. Source data are provided as a Source Data file.

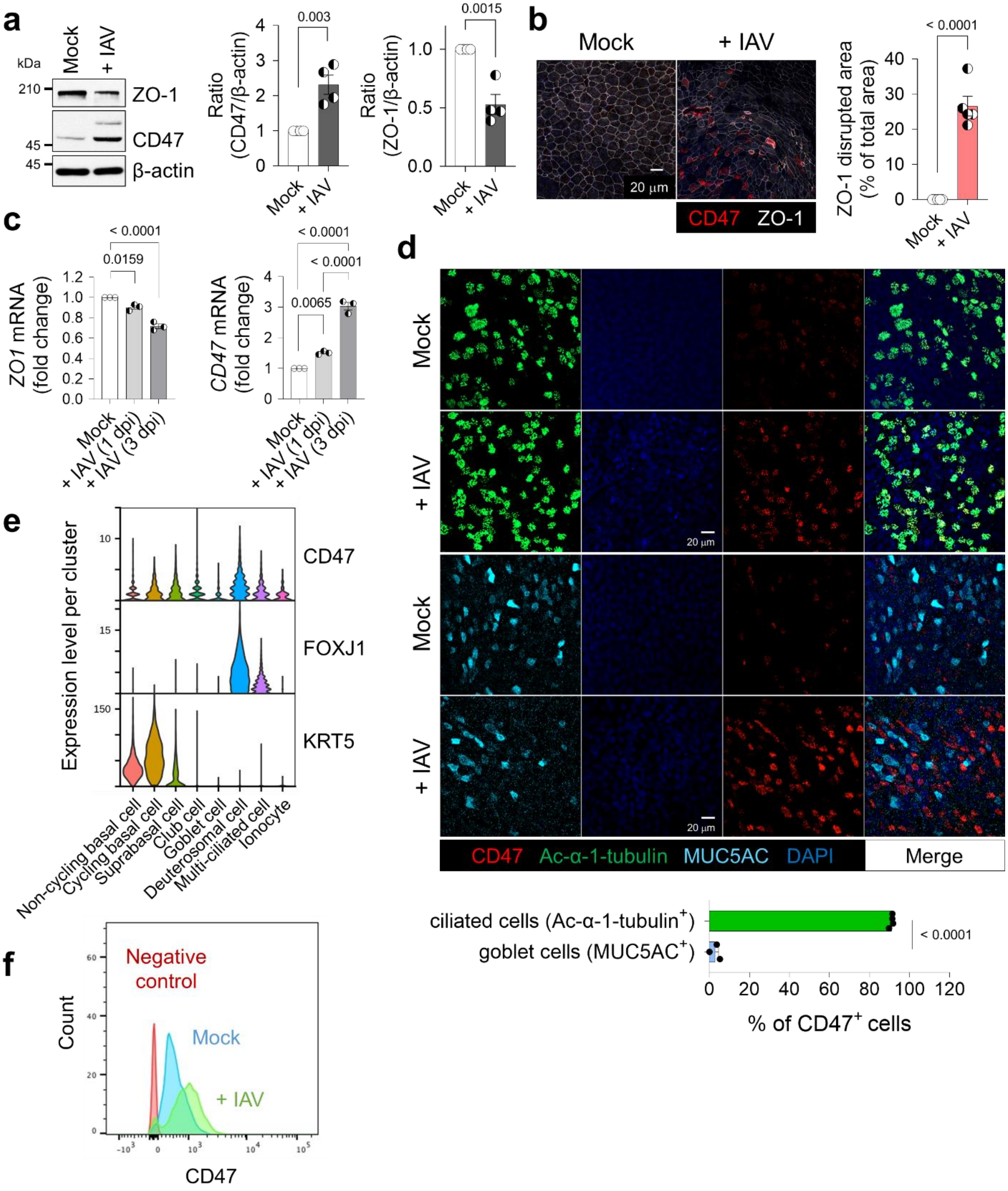

Supplementary Fig. 2. CD47 induction and exposure in the apical surface of ciliated HNECs following influenza virus infection. HNECs were infected with (+ *IAV*) or without (*Mock*) influenza virus. **a** Immunoblot analysis of junction protein Zona occluden-1 (ZO-1) and surface protein CD47 at 1 dpi. Normalized CD47 protein levels are

presented as bar graphs (n = 4). **b** Representative whole-mount images of ZO-1 (white) and CD47 (red) at 1 dpi. The area where ZO-1 disconnection occurred are depicted as bar graphs (n = 5). **c** Quantitative PCR (qPCR) analysis of *ZO-1* and *CD47* mRNAs at 1 and 3 dpi (n = 3). **d** Whole-mount images of influenza virus-infected HNECs. Co-staining of CD47 (red) and ciliated cell-specific marker protein Ac-α-tubulin (green) or goblet cell-specific marker protein MUC5AC (cyan). Percentages of CD47-positive cells were presented as bar graphs ($n \geq 3$). **e** Expression of *CD47*, ciliated cell-specific marker *FoxJ1*, and basal cell-specific marker *KRT5* are presented as violin graphs based on the scRNA-seq of ALI-cultured HNECs (14 days). **f** Flow cytometry analysis of CD47 expression in the surface of HNECs at 1 dpi. Data are presented as mean values ± SEM. Significance was determined by unpaired two-tailed Student's t test or one-way ANOVA with Tukey's multiple comparisons test. Source data are provided as a Source Data file.

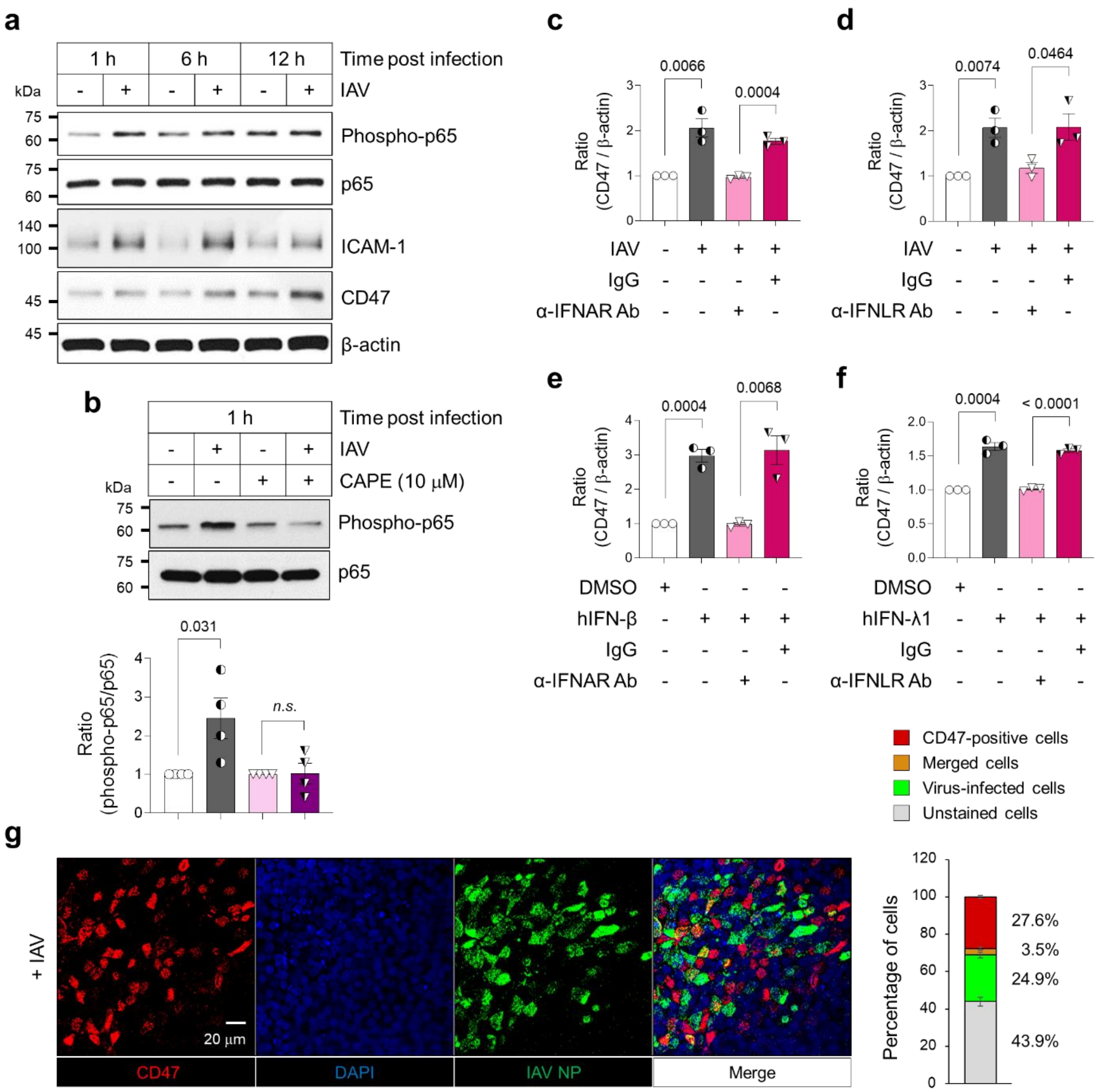

Supplementary Fig. 3. Viral infection-induced CD47 expression via NF-κB/IFN signaling pathway. a HBECs were infected with influenza virus and harvested at 1, 6, and 12 h after viral infection. Note that these blots represent one of three independent experiments. **b** HBECs were treated with or without 10 μM of NF-κB inhibitor caffeic acid phenethyl ester (CAPE) for 1 h before influenza virus infection. Immunoblot analysis of phospho-p65, p65, ICAM-1, and CD47 in the indicated time (*n* = 4). **c-f** HBECs were infected with influenza virus (**c** and **d**), and with recombinant human IFN-β (**e**) or IFN-λ1 (**f**). CD47 protein expression was analyzed by immunoblotting after pretreating the cells with either IgG or α-IFNAR Ab (**c** and **e**) or α-IFNLR Ab (**d** and **f**) (*n* = 3). **g** Whole-mount

images of influenza virus-infected HBECs. Co-staining of CD47 (red) and the viral protein NP (green). Percentage of CD47-positive cells, virus-infected cells, merged cells and unstained cells were presented as bar graphs (*n* = 3). Data are presented as mean values ± SEM. Significance was determined by unpaired two-tailed Student's *t* test. *n.s.*, not significant. Source data are provided as a Source Data file.

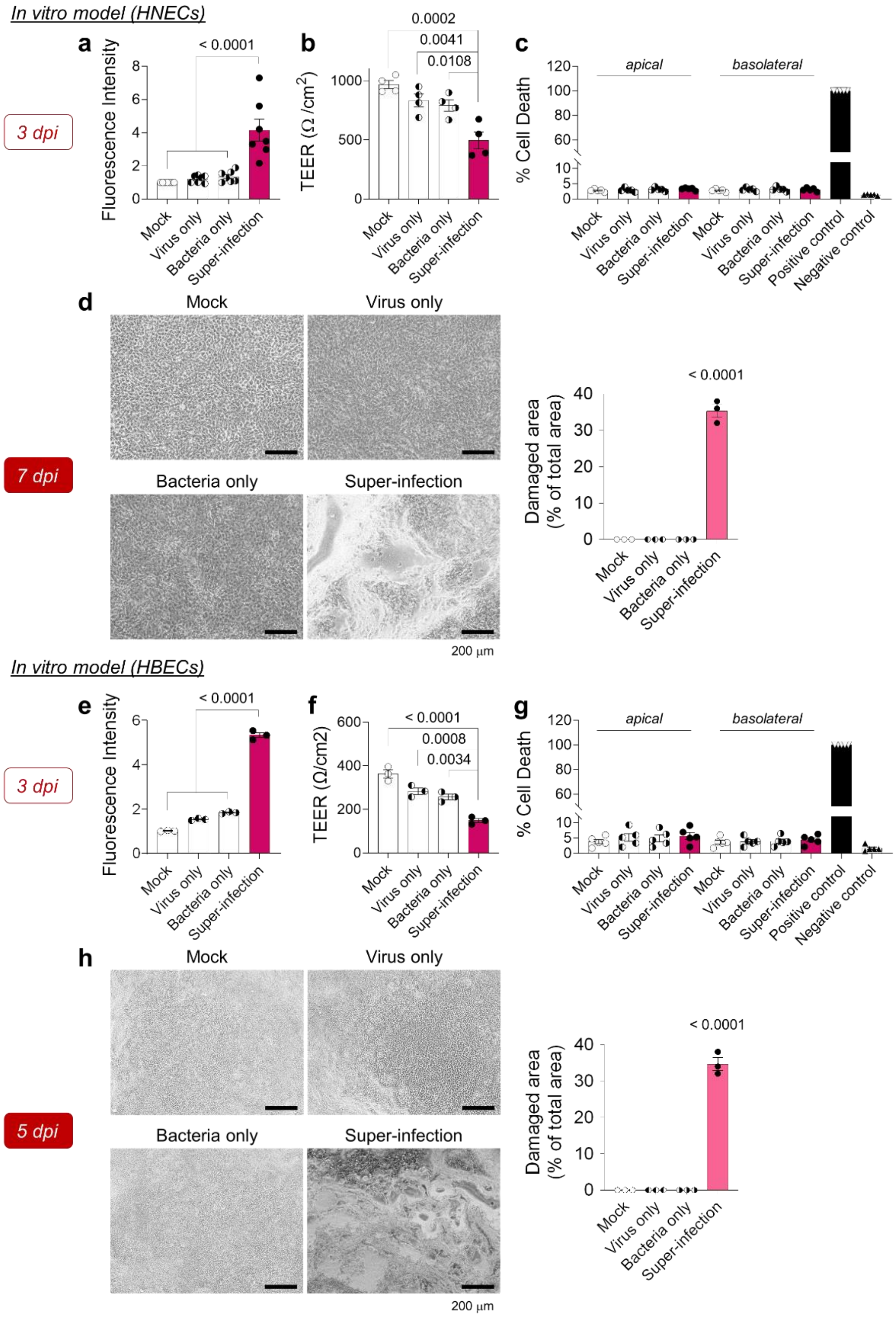

Supplementary Fig. 4. *In vitro* model of super-infection used in this study. a-h *In vitro* super-infection models in HNECs (**a**-**d**) and HBECs (**e**-**h**). The ratio of paracellular

FITC-dextran permeability (**a** and **e**), trans-epithelial electrical resistance (**b** and **f**), and LDH levels of apical or basolateral fractions (**c** and **g**) measured in non-infected (*Mock*), influenza virus-infected (Virus only, MOI 1), *S. aureus*-infected (Bacteria only, MOI 5 for HNECs or MOI 3 for HBECs), and viral-bacterial co-infected cells (Super-infection) ($n \geq 3$). Microscopic images show morphological changes of HNECs at 7 dpi (**d**) and HBECs at 5 dpi (**h**). The percentage of the damaged area is presented as bar graphs ($n = 3$). Data are presented as mean values ± SEM. Significance was determined by one-way ANOVA with Tukey's multiple comparisons test. Source data are provided as a Source Data file.

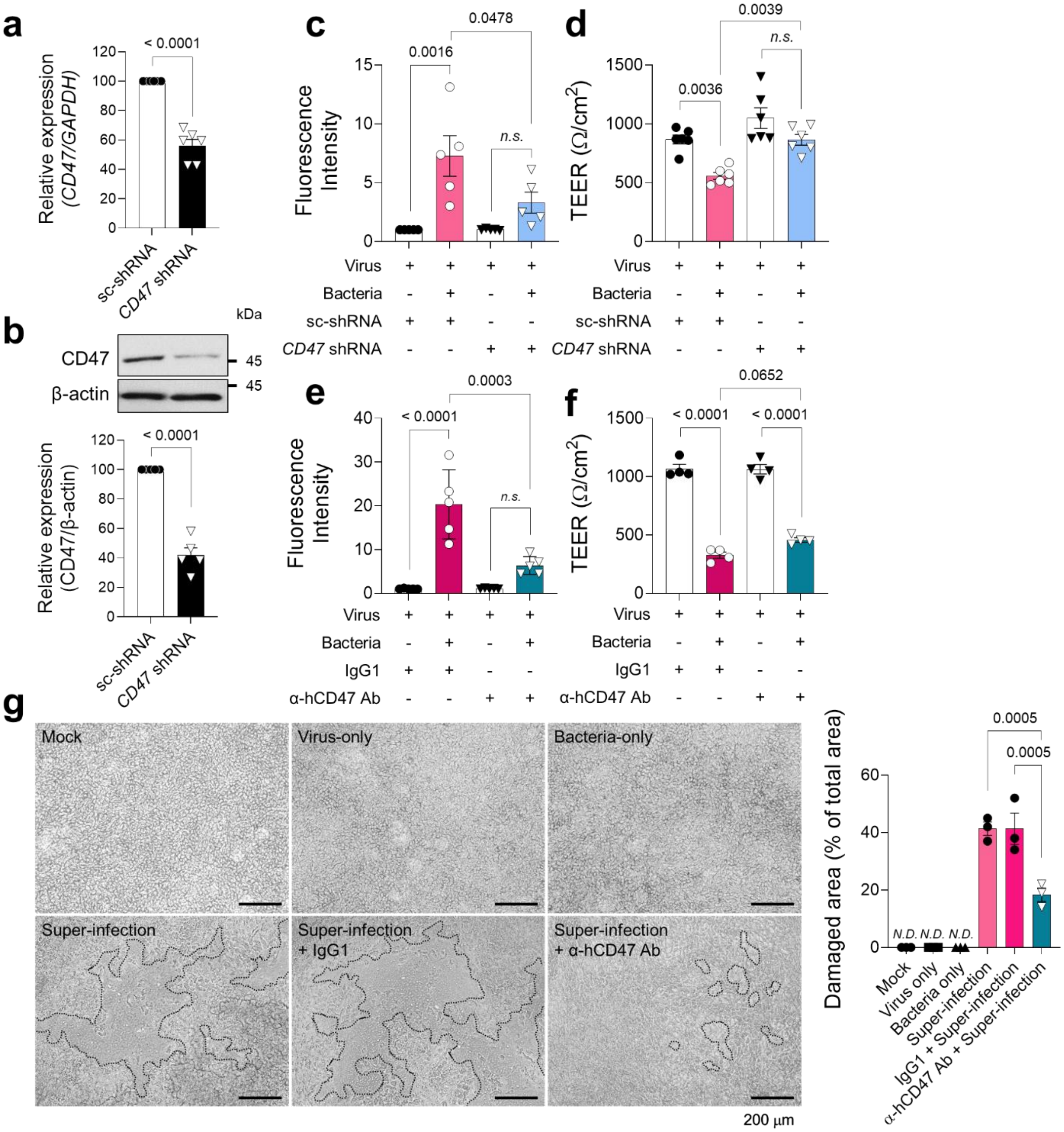

Supplementary Fig. 5. Protective effect of knock-down and neutralization of CD47 on super-infection in HNECs. For *CD47* knock-down, HNECs were transfected with scrambled shRNA (*sc-shRNA*) or shRNA targeting *CD47* (*CD47 shRNA*) using a lentiviral delivery system. For CD47 neutralization, HNECs were treated with either IgG1 (MOPC-21) or α-hCD47 (B6.H12.2) antibodies. **a** Gene expression of *CD47* was analyzed using qRT-PCR (normalized by *18s* mRNA) ($n = 6$). **b** Protein expression of CD47 was analyzed via immunoblotting (normalized by β-actin). Normalized CD47 protein levels are

presented as bar graphs (*n* = 5). **c**-**f** Paracellular FITC-dextran permeability (*n* = 5) (**c**, **e**) and trans-epithelial resistance (*n* ≥ 4) (**d**, **f**) of virus only and super-infection groups were measured in the presence of either *CD47* shRNA or α-hCD47 antibodies. **g** Microscopic images of HNECs at 7 dpi. The damaged area is depicted in dotted lines and the percentage of the damaged area is presented as bar graphs (*n* = 3). Data are presented as mean values ± SEM. Significance was determined by unpaired two-tailed Student's *t* test or one-way ANOVA with Tukey's multiple comparison test. *n.s.*, not significant. Source data are provided as a Source Data file.

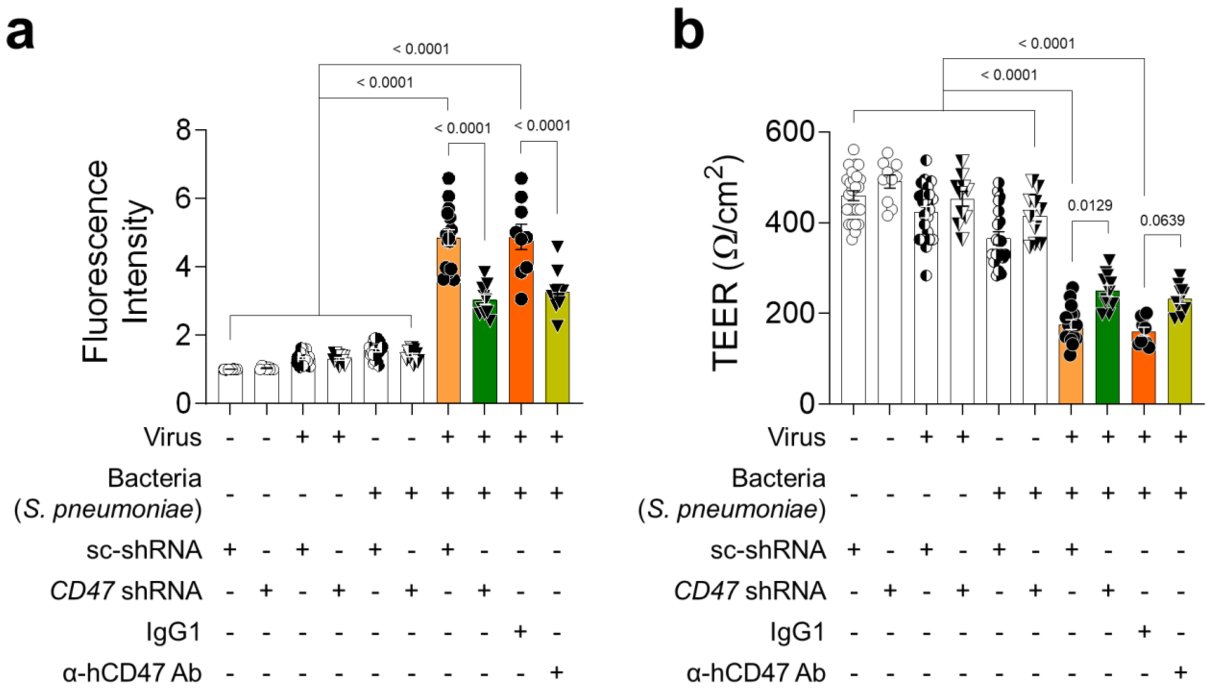

Supplementary Fig. 6. Protective effect of knock-down and neutralization of CD47 on the *S. pneumoniae*-mediated super-infection in HBECs. a, **b** For *CD47* knock-down, HBECs were transfected with scrambled shRNA (*sc-shRNA*) or shRNA targeting *CD47* (*CD47 shRNA*) using a lentiviral delivery system. For CD47 neutralization, HBECs were treated with either IgG1 (MOPC-21) or α-hCD47 (B6.H12.2) antibodies. Paracellular FITC-dextran permeability (**a**) and trans-epithelial resistance (**b**) of virus-only, bacteria-only, and super-infection groups were measured in the presence of sc-shRNA, *CD47* shRNA, IgG1, or α-hCD47 antibodies ($n \geq 10$). Data are presented as mean values ± SEM. Significance was determined by one-way ANOVA with Tukey's multiple comparisons test. Source data are provided as a Source Data file.

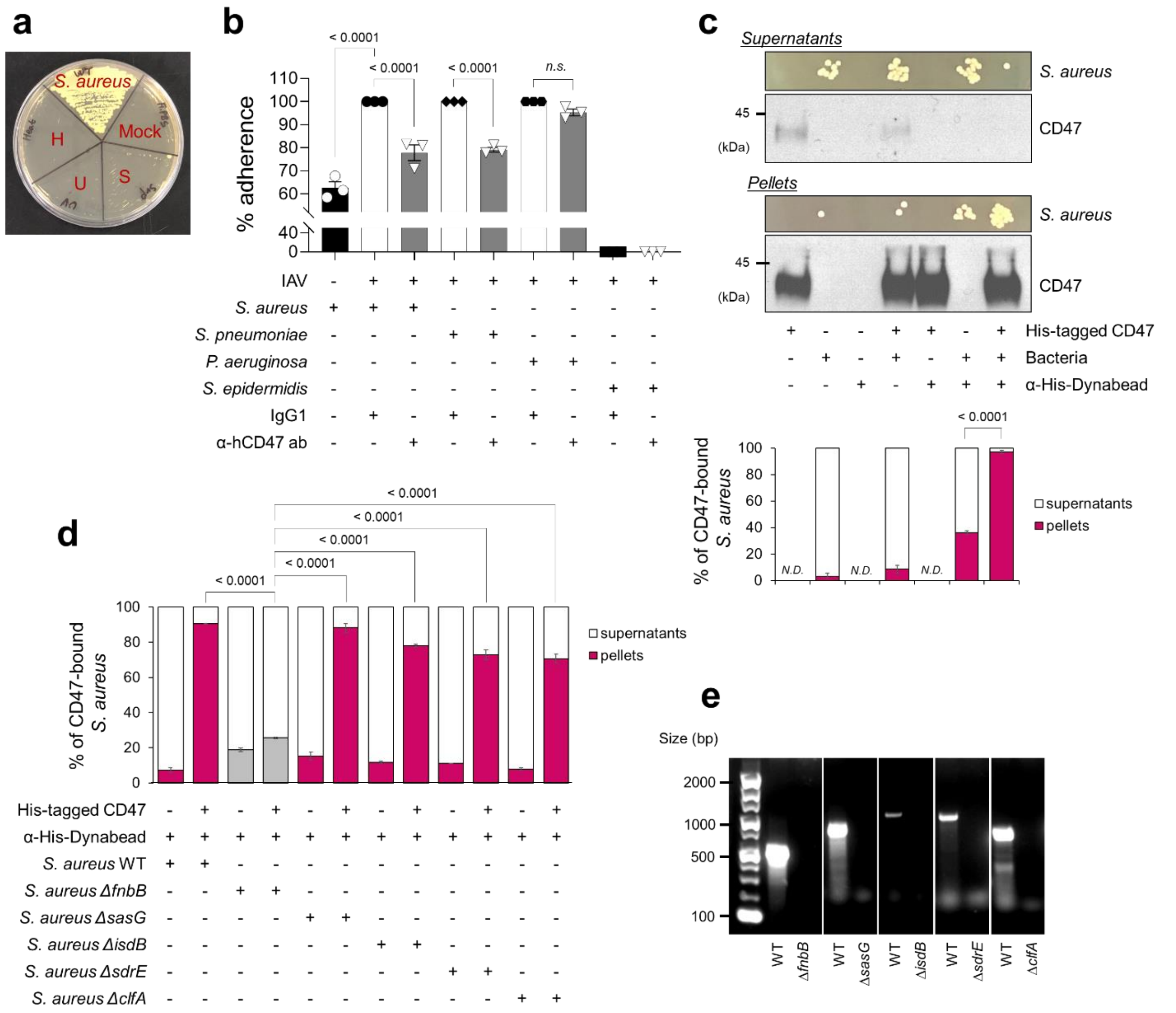

Supplementary Fig. 7. Direct interactions between epithelial CD47 and *S. aureus*. a Bacterial plating of live *S. aureus* or *S. aureus*-derived samples (*S*, supernatant of *S. aureus*-cultured media; *U*, UV-killed *S. aureus*; *H*, heat-killed *S. aureus*). **b** Bacterial adhesion assay. Adherence of *S. aureus* (gram-positive), *S. pneumoniae* (gram-positive), *P. aeruginosa* (gram-negative), and *S. epidermidis* (gram-positive, commensal bacterium), was assessed in the influenza-infected A549 cells. The cells were treated with IgG1 control antibodies or α-hCD47 neutralizing antibodies (2 h), followed by bacterial infection (MOI 10) for 3 h (*n* = 3). **c** Pull-down assay using His-tagged hCD47 recombinant protein. Bacterial plating and immunoblot analysis were performed using supernatants and pellets after separation with α-His-Dynabeads™/DynaMag™-2 system. The graphs present the percentage of colony numbers grown in the culture of the supernatants or the pellets (*n* = 3). **d** *in vitro* pull-down assay of WT and five cell wall-

anchored proteins (CWAs) deletion mutants of *S. aureus*; *fnbB::Tn* (*ΔfnbB*), *sasG::Tn* (*ΔsasG*), *isdB::Tn* (*ΔisdB*), *sdrE::Tn* (*ΔsdrE*), and *clfA::Tn* (*ΔclfA*) (n = 3). **e** Gene deletions were validated by PCR. Data are presented as mean values ± SEM. Significance was determined by unpaired two-tailed Student's t test or one-way ANOVA with Tukey's multiple comparisons test. *n.s.*, not significant; *N.D.*, not determined; bp, base pair. Source data are provided as a Source Data file.

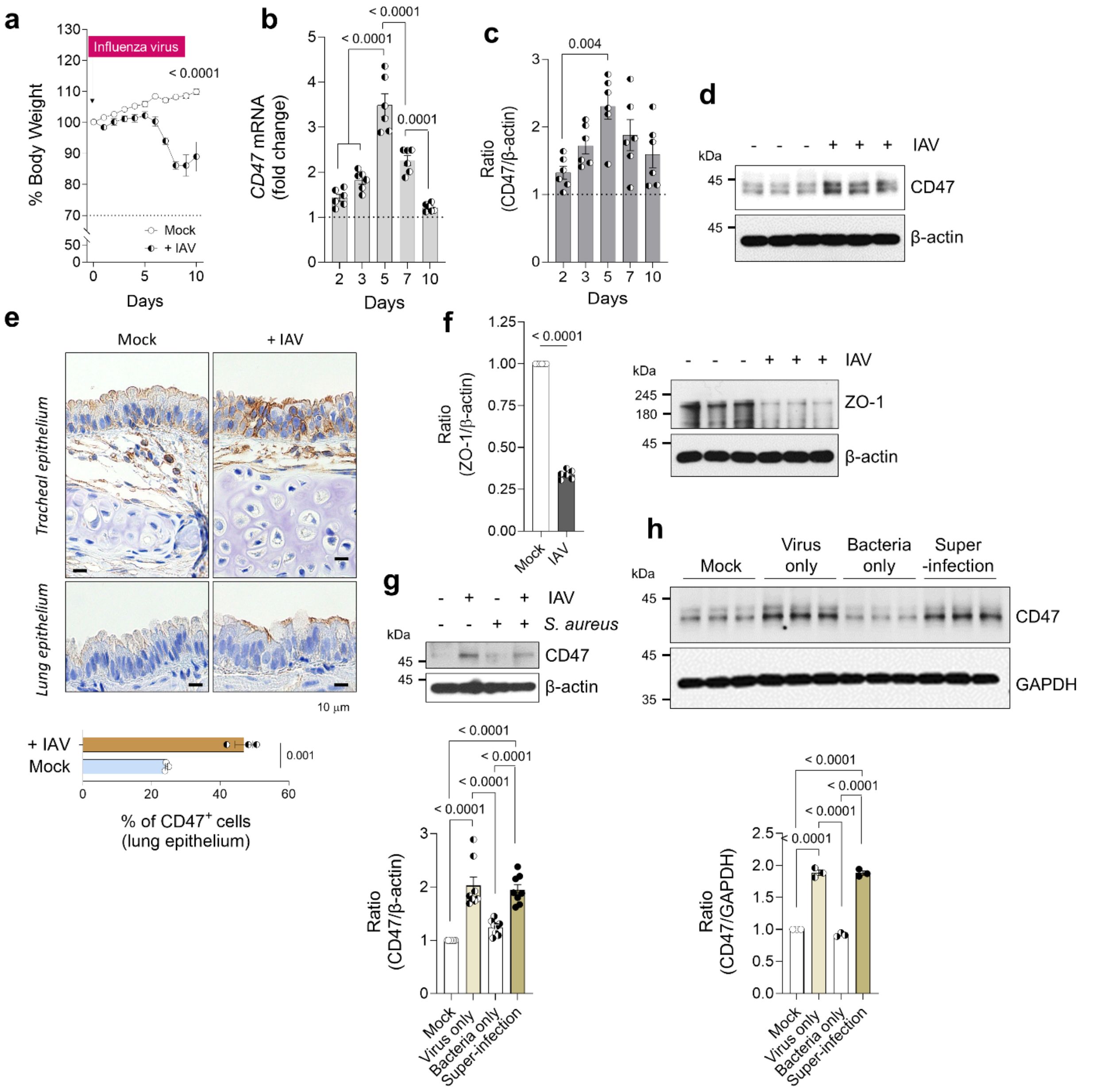

Supplementary Fig. 8. Induction of CD47 upon viral infection. a-f 6–8-weeks-old (18–21 g of body weight) C57BL/6 WT mice were infected with influenza virus (100 PFU). Body weight loss was monitored in two groups of mice for 10 days; non-infected (*Mock*) and influenza virus-infected (*+ IAV*) (**a**). The dotted line indicates the body weight exclusion cut-off. Changes in *CD47* mRNAs (n = 6) (**b**) and CD47 proteins (n = 6) (**c**) were analyzed at 2, 3, 5, 7 and 10 dpi. Representative immunoblots of CD47 at 5 dpi (**d**). Cross section of tracheal epithelium (*upper panels*) and lung epithelium (*lower panels*) at 7 dpi were stained with antibodies against CD47 and visualized via 3, 3'-diaminobenzidine tetrahydrochloride (DAB) staining (**e**). The slides were counterstained with hematoxylin.

Percentages of CD47-positive cells were presented as bar graphs (*n* = 3). Changes in ZO-1 proteins were analyzed at 5 dpi. Normalized ZO-1 protein levels are presented as bar graphs (*n* = 6) (**f**). **g**-**h** Changes in CD47 proteins were analyzed in HBECs (*n* = 6) (**g**) or four groups of control floxed (*Cd47*$^{f/f}$) mice (whole lung lysates, *n* = 3) (**h**); non-infected (*Mock*), influenza virus-infected (*Virus only*), *S. aureus*-infected (*Bacteria only*), and viral–bacterial co-infected mice (*Super-infection*). Normalized CD47 protein levels are presented as bar graphs. Data are presented as mean values ± SEM. Significance was determined by unpaired two-tailed Student's *t* test, one-way ANOVA with Tukey's multiple comparisons test, or two-way ANOVA with Tukey's multiple comparisons test (% Body weight). Source data are provided as a Source Data file.

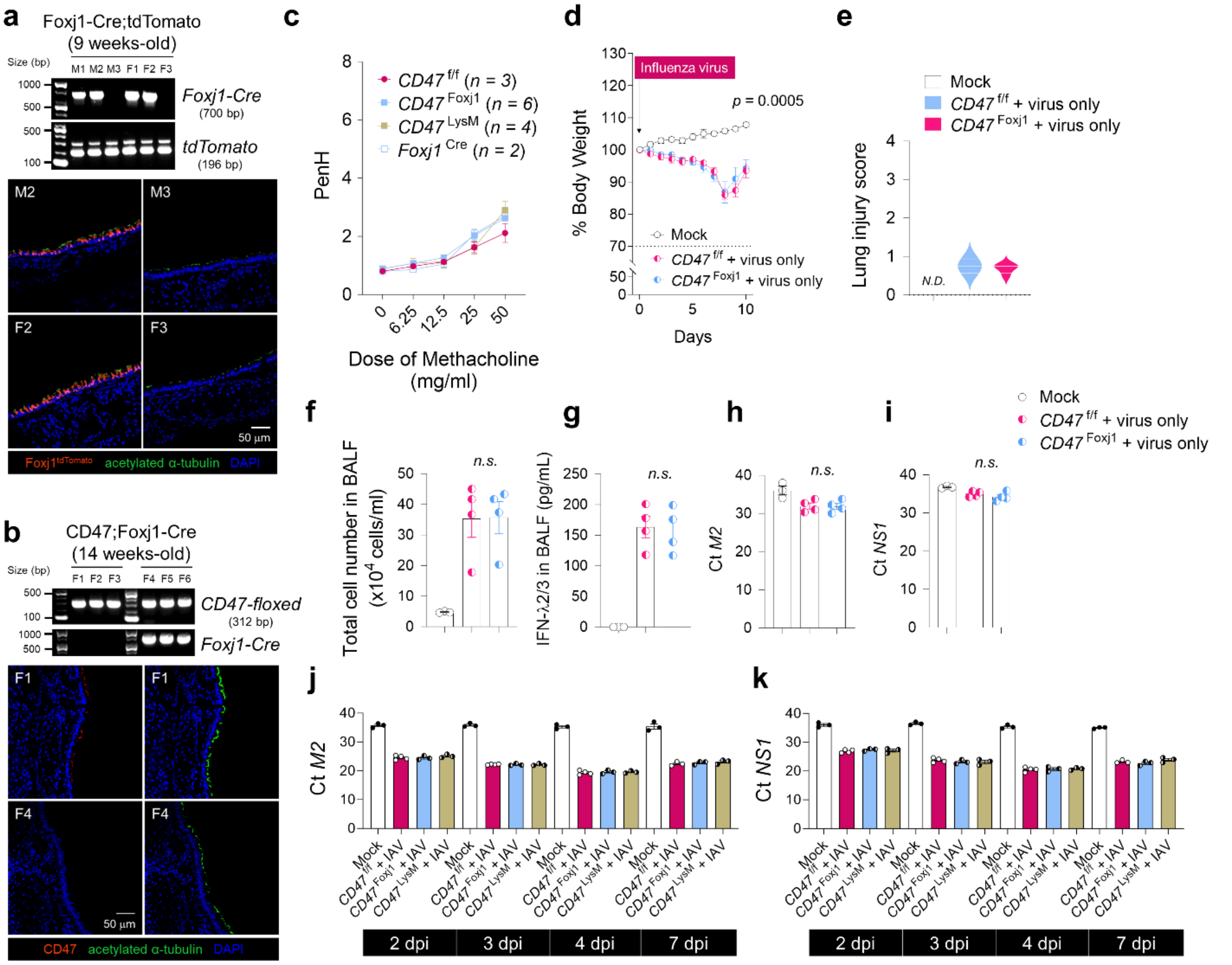

Supplementary Fig. 9. Characterization of tissue-specific CD47 deletion mice. a Specificity of *Foxj1*-Cre was validated using *Foxj1*-Cre;tdTomato mice (trachea). PCR amplification of mice genomic DNA using the specific primers for *Foxj1*-Cre and *tdTomato* (*upper panels*). *Foxj1*^{tdTomato}-expressing cells (red) were co-stained with ciliated cell-specific marker protein Ac-α-tubulin (green) (*lower panels*). **b** *Foxj1*^{Cre}-specific CD47 deletion was determined in the lower airway epithelium (trachea). PCR amplification of mice genomic DNA using the specific primers for *CD47-floxed* cassette and *Foxj1*-Cre (*upper panels*). CD47-expressing cells (red) and ciliated cell-specific marker protein Ac-α-tubulin (green) are shown on the same slide separately (*lower panels*). **c** Airway hyper-responsiveness (AHR) is presented as Penh in response to methacholine. **d**-**i** 6–8-weeks-old (18–21 g of body weight) *FoxJ1*-Cre;floxed (*Cd47*$^{\mathrm{FoxJ1}}$) mice and control floxed (*Cd47*$^{\mathrm{f/f}}$) mice were infected with influenza virus (100 PFU). Body weight loss was monitored in two groups of mice for 10 days ($n \geq 3$) (**d**). Lung injury scores are presented as violin plots ($n \geq 3$) (**e**). Total cell number in BAL fluids (BALF) (**f**) and levels of type III interferons

(IFN-λ2/3) were measured at 10 dpi ($n \geq 3$) (**g**). **h**-**i** Mouse lungs were harvested at 10 dpi for qPCR analysis to evaluate the transcriptional level of viral *M2* (**h**) and *NS1* (**i**) genes in virus-infected $CD47^{\mathrm{f/f}}$ and $CD47^{\mathrm{Foxj1}}$ compared with Mock ($n \geq 3$). **j**-**k** Mouse lungs were harvested at 2, 3, 4 and 7 dpi for qPCR analysis to evaluate the transcriptional level of viral *M2* (**j**) and *NS1* (**k**) genes in virus-infected $CD47^{\mathrm{f/f}}$, $CD47^{\mathrm{Foxj1}}$, and $CD47^{\mathrm{LysM}}$ mice compared with Mock ($n \geq 3$). Data are presented as mean values ± SEM. Significance was determined by one-way ANOVA with Tukey's multiple comparisons test. *M*, male; *F*, female; bp, base pair; *N.D.*, not detected; *n.s.*, not significant. Source data are provided as a Source Data file.

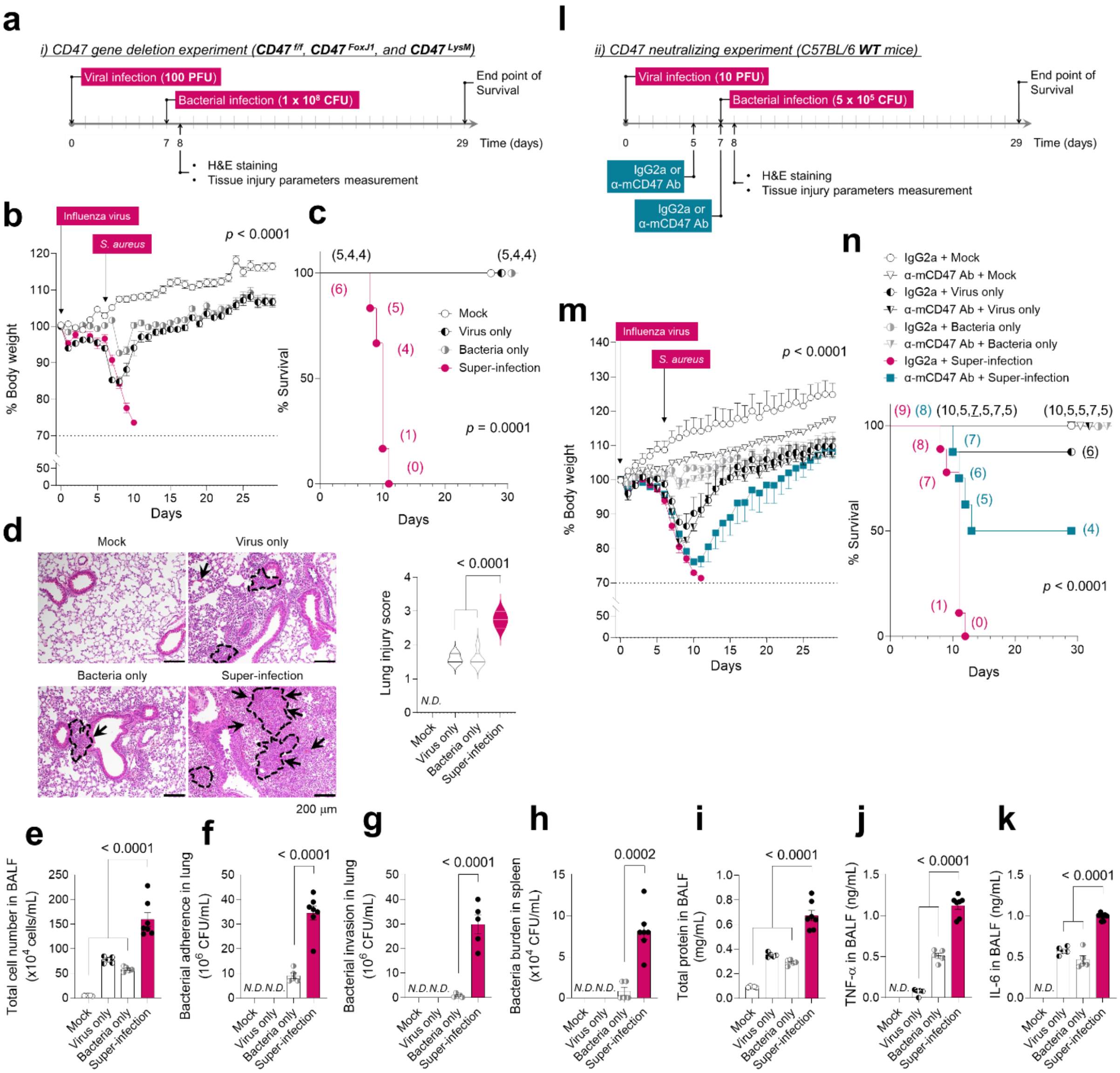

Supplementary Fig. 10. Two *in vivo* models of super-infection used in this study. a-k For CD47 gene deletion experiment (**a**), 6–8-weeks-old (18–21 g of body weight) control floxed (*Cd47* $^{f/f}$) mice were infected with 100 PFU of influenza virus on day 0 and with 1 x 10^8 CFU of *S. aureus* on day 7. Body weight loss (**b**) and survival rates (**c**) were monitored in four groups of mice for 29 days; non-infected (*Mock*, *n* = 5), influenza virus-infected (*Virus only*, *n* = 4), *S. aureus*-infected (*Bacteria only*, *n* = 4), and viral bacterial co-infected mice (*Super-infection*, *n* = 6). Representative hematoxylin and eosin (H&E) staining of lung sections in four groups (**d**). The dotted lines indicate lymphocytic infiltration and arrows indicate alveolar hemorrhage. Lung injury scores are presented as violin plots. Tissue injury parameters were measured at 24 h after bacterial infection: total

cell number in BAL fluids (**e**), bacterial adherence (**f**) and invasion (**g**) in the lung, and bacterial burden in the spleen (**h**), total protein concentrations in BAL fluids (**i**), and inflammatory cytokines TNF-α (**j**) and IL-6 (**k**) in BAL fluid ($n \geq 5$). **l**-**n** For CD47 neutralizing experiment (**l**), 6–8-weeks-old (18–21 g of body weight) C57BL/6 WT mice were infected with 10 PFU of influenza virus on day 0 and with 5 x 10^5 CFU of *S. aureus* on day 7. Before bacterial infection, mice were intranasally treated twice with IgG2a control antibodies (2A3) or α-mCD47 neutralizing antibodies (MIAP301) at 5 and 7 dpi. Body weight loss (**m**) and survival rate (**n**) were monitored in eight groups of mice for 29 days; *IgG2a* + *Mock* (*n* = 10), *α-mCD47 Ab* + *Mock* (*n* = 5), *IgG2a* + *Virus only* (*n* = 8), *α-mCD47 Ab* + *Virus only* (*n* = 5), *IgG2a* + *Bacteria only* (*n* = 8), *α-mCD47 Ab* + *Bacteria only* (*n* = 5), *IgG2a* + *Super-infection* (*n* = 9), and *α-mCD47 Ab* + *Super-infection* (*n* = 8). The dotted line indicates the body weight exclusion cut-off. A mantel cox survival analysis was used to compare the survival rates between groups. The numbers in parenthesis represent the count of surviving mice. Data are presented as mean values ± SEM. Significance was determined by one-way ANOVA with Tukey's multiple comparisons test or two-way ANOVA with Tukey's multiple comparisons test (% Body weight). *N.D.*, not detected. Source data are provided as a Source Data file.

Supplementary Table 1. Primers used for RT-qPCR and genotyping

Gene name	Forward primer sequence (5' to 3')	Reverse primer sequence (5' to 3')
Human *CD47*	tcggtcctgcctgtaacg	taccaggggccacatctc
Human *ZO-1*	tggtgtcctacctaattcaactca	cgccagctacaaatattccaaca
Human *ICAM-1*	ccttcctcaccgtgtactgg	agcgtagggtaaggttcttgc
Human *CEACAM5*	aggccaataactcagccagt	gggtttggagttgttgctgg
Human *PAFR*	accagggcaactggatactc	aaggccacagagcagtaggt
Human *Integrin α3*	aagggaccttcaggtgca	tgtagccggtgatttaccat
Human *Integrin β1*	gaagggttgccctccaga	gcttgagcttctctgctgtt
Human *Interferon β1*	attctaactgcaacctttcg	gttgtagctcatggaaagag
Human *Interferon λ1*	gactttggtgctaggcttgg	agatttgaacctgccaatgtg
Human *Interferon λ2/3*	gccaaagatgccttagaagag	cagaaccttcagcgtcagg
Human *Mx1*	agagaaggtgagaagctgatcc	ttcttccagctccttctctctg
Human *Oas1*	gctcctaccctgtgtgtgtgt	tggtgagaggactgaggaaga
Mouse *CD47*	aggatggcgtgagggagagc	ctcttattcgtatggctg
Influenza virus *M2*	aagaccaatcctgtcacctctga	caaagcgtctacgctgcagtcc
Influenza virus *NS1*	agcaaaagcagggtgaca aagaca	tcggtgaaagccctta
Δ *fnbB*	cgcagtgttgagataccatgag	ggaaagtgggagttcagctactg
Δ *sdrE*	gctttgcagttgcacaaccagc	ctttgaggcaattgttgatcagag
Δ *clfA*	gcaatcaaatcgtacgttgtcg	gtagtagcttcaccagttaccgg
Δ *isdB*	ggttgtgcgaattccattaatgtg	tgtcaaatggcgaagcacaagc
Δ *sasG*	ctctgcgttatgagtgactgacg	gtgttatattccattgtgcaactcc
18s	gcttaatttgactcaacacggga	agctatcaatctgtcaatcctgtc
GAPDH	caatgacccсttcattgacc	gacaagcttcccgttctcag
Ppia	cgcttgctgcagccatggtc	cagctcgaaggagacgcggc

Genotype	Forward primer sequence (5' to 3')	Reverse primer sequence (5' to 3')
tdTomato Wildtype	aagggagctgcagtggagta	ccgaaaatctgtgggaagtc
tdTomato Mutant	ctgttcctgtacggcatgg	ggcattaaagcagcgtatcc
LysM-Cre Wildtype	ttacagtcggccaggctgac	cttgggctgccagaatttctc
LysM-Cre Mutant	cccagaaatgccagattacg	cttgggctgccagaatttctc
Foxj1-Cre	gcctgcattaccggtcgatgcaacga	gtggcagatggcgcggcaacaccatt
CD47-floxed	tctacactaaactcagctggcctgg	ctgtctctgtgctctctggctaagg
