# Supplementary material for: Airway epithelial CD47 plays a critical role in inducing influenza virus-mediated bacterial super-infection
Source: Nat Commun. 2024 Apr 30;15:3666. doi: 10.1038/s41467-024-47963-5 (PMC11063069; doi:10.1038/s41467-024-47963-5)
Supplement: Supplementary file 3 — Reporting Summary [file 41467_2024_47963_MOESM3_ESM.pdf]

nature portfolio

Corresponding author(s): Ji-Hwan Ryu

Last updated by author(s): Mar 29, 2024

Reporting Summary

Nature Portfolio wishes to improve the reproducibility of the work that we publish. This form provides structure for consistency and transparency in reporting. For further information on Nature Portfolio policies, see our Editorial Policies and the Editorial Policy Checklist.

Statistics

For all statistical analyses, confirm that the following items are present in the figure legend, table legend, main text, or Methods section.

n/a	Confirmed	
☐	☒	The exact sample size (*n*) for each experimental group/condition, given as a discrete number and unit of measurement
☐	☒	A statement on whether measurements were taken from distinct samples or whether the same sample was measured repeatedly
☐	☒	The statistical test(s) used AND whether they are one- or two-sided<br>*Only common tests should be described solely by name; describe more complex techniques in the Methods section.*
☒	☐	A description of all covariates tested
☐	☒	A description of any assumptions or corrections, such as tests of normality and adjustment for multiple comparisons
☐	☒	A full description of the statistical parameters including central tendency (e.g. means) or other basic estimates (e.g. regression coefficient) AND variation (e.g. standard deviation) or associated estimates of uncertainty (e.g. confidence intervals)
☐	☒	For null hypothesis testing, the test statistic (e.g. *F*, *t*, *r*) with confidence intervals, effect sizes, degrees of freedom and *P* value noted<br>*Give P values as exact values whenever suitable.*
☒	☐	For Bayesian analysis, information on the choice of priors and Markov chain Monte Carlo settings
☒	☐	For hierarchical and complex designs, identification of the appropriate level for tests and full reporting of outcomes
☒	☐	Estimates of effect sizes (e.g. Cohen's *d*, Pearson's *r*), indicating how they were calculated

Our web collection on statistics for biologists contains articles on many of the points above.

Software and code

Policy information about availability of computer code

Data collection	We did not use any unpublished code to collect the data in this study. Confocal images were acquired by LSM980 (Carl Zeiss) confocal microscopy. PCR reaction was performed using QuantStudio 3 Real-Time PCR System (Thermo Scientific). Flow cytometry was performed using the BD FACSLyric™ (BD Biosciences). Airway hyper-responsiveness (AHR) was measured as the change in airway function using whole-body plethysmography (Buxco Electronics Ltd., USA). The sample absorbance used in enzyme-linked immunosorbent assay (ELISA) was measured at 450 nm using a VersaMax™ Microplate Reader (Molecular Devices).
Data analysis	We did not use any unpublished code to collect the data in this study. Analyses and processing of confocal images were performed on ZEN image software (Carl Zeiss, ZEN 3.0 lite). Proteomic analysis was performed using DAVID software v6.8 (https://david.ncifcrf.gov/tools.jsp). All statistical analyses were performed using Prism GraphPad 10.1.0. software. Data obtain from flow cytometry was acquired with BD FACSuite v1.3 (BD Biosciences) and analyzed with FlowJo™ v.10.6.2 software (BD Biosciences). Data obtained from Airway hyper-responsiveness (AHR) was analyzed using FinePoint software (Buxco Electronics Ltd.). Data obtained from enzyme-linked immunosorbent assay (ELISA) was analyzed with SoftMax® Pro Software v5.2 (Molecular Devices).

For manuscripts utilizing custom algorithms or software that are central to the research but not yet described in published literature, software must be made available to editors and reviewers. We strongly encourage code deposition in a community repository (e.g. GitHub). See the Nature Portfolio guidelines for submitting code & software for further information.

Data

Policy information about availability of data

All manuscripts must include a data availability statement. This statement should provide the following information, where applicable:

- Accession codes, unique identifiers, or web links for publicly available datasets
- A description of any restrictions on data availability
- For clinical datasets or third party data, please ensure that the statement adheres to our policy

Data presented in the manuscript will be made available to investigators following request. The raw data files of the iTRAQ dataset are available in the ProteomeXchange Consortium via the PRIDE partner repository under accession code PXD042618 (http://proteomecentral.proteomexchange.org). Proteomics data analysis was performed using the Database for Annotation, Visualization and Integrated Discovery (DAVID) v6.8 (https://david.ncifcrf.gov/tools.jsp). All relevant data are included in the manuscript and the Supplementary Information. Source data are provided with this paper.

Research involving human participants, their data, or biological material

Policy information about studies with human participants or human data. See also policy information about sex, gender (identity/presentation), and sexual orientation and race, ethnicity and racism.

Reporting on sex and gender	Human nasal polyp tissues from chronic rhinosinusitis (CRS) patients (n = 43) were collected in the department of Otorhinolaryngology at Severance Hospital in Yonsei University College of Medicine (IRB no. 4-2016-1153 and 4-2021-0573). Among 43 patients, the number of male is 25 and the number of female is 18. Since we focused on CD47 expression in human nasal epithelial cells derived from nasal polyp tissues which obtained during the endoscopic sinus surgery, sex- and gender-based analysis were not performed.
Reporting on race, ethnicity, or other socially relevant groupings	All patients were East Asian (Korean).
Population characteristics	Forty-three patients (18 females and 25 males) with chronic rhinosinusitis (CRS) were enrolled in this study. Each patient met the criteria for CRS as defined by the European Position Paper on Rhinosinusitis and Nasal Polyps 2020 guidelines. The median age of the patients was 52 years (interquartile rage 28.5). Nasal polyp tissues were obtained during the endoscopic sinus surgery and used for the isolation of human nasal epithelial cells.
Recruitment	This study recruited 43 patients with CRS who underwent endoscopic sinus surgery in Severance Hospital from November 2020 to April 2023. Informed consent was obtained from all donors, and this study was conducted according to the principles of the Declaration of Helsinki. All participants were diagnosed with CRS, as defined by the European Position Paper on Rhinosinusitis and Nasal Polyps 2020; underwent endoscopic sinus surgery; and having Nasal polyps. The inclusion criteria were as follows: (a) patients diagnosed with CRS, as defined by the European Position Paper on Rhinosinusitis and Nasal Polyps 2020; (b) those who underwent endoscopic sinus surgery; and (c) those who agreed to participate in the study. Patients who satisfied all of the above criteria were included in the study.
Ethics oversight	All experiments using human nasal epithelial cells (HNECs) were approved by the institutional review board of Yonsei University College of Medicine (4-2016-1153 and 4-2021-0573), and all participants provided informed consent.

Note that full information on the approval of the study protocol must also be provided in the manuscript.

Field-specific reporting

Please select the one below that is the best fit for your research. If you are not sure, read the appropriate sections before making your selection.

☒ Life sciences ☐ Behavioural & social sciences ☐ Ecological, evolutionary & environmental sciences

For a reference copy of the document with all sections, see nature.com/documents/nr-reporting-summary-flat.pdf

Life sciences study design

All studies must disclose on these points even when the disclosure is negative.

Sample size	No statistical method was used to predetermine the sample size. Throughout the study, at least three samples were included for one experiment to ensure sufficient reproducibility of the results. Biological replicates (n) and the numbers of independent experiment are indicated in the figure legends. At least 3 biological samples were included for one experiment and 1 to 3 independent experiments were performed to ensure sufficient reproducibility of the results.
Data exclusions	In the super-infection mouse model, mice were monitored daily for changes in weight and mortality until day 29. Mice manifesting a weight loss exceeding 30% of their initial body weight were humanely euthanized and subsequently excluded from further analysis in both weight loss and survival data, in accordance with the humane euthanasia criteria defined by the relevant animal protocols. All animal work was approved by the Institutional Animal Care and Use Committee (IACUC) at Yonsei University College of Medicine (protocol number 2022-0257), according to guidelines outlined by the Association for Assessment and Accreditation of Laboratory Animal Care (AAALAC) International

(facility number 001071).

Replication: Each experiments was replicated at least three times. The number of replicates is stated in figure legends.

Randomization: Mice allocation was randomized in all in vivo experiments. However, since randomization is not relavant to experiments other than those involving mice, such as in this in vitro basic science study, no randomization steps were conducted for experiments or data analysis.

Blinding: The investigators were not blinded to allocation during the experiments and outcome assessment. Data collection and analysis were not blinded due to the nature of the experimental designs in this study, but unbiased quantifications were applied to obtain the results.

Reporting for specific materials, systems and methods

We require information from authors about some types of materials, experimental systems and methods used in many studies. Here, indicate whether each material, system or method listed is relevant to your study. If you are not sure if a list item applies to your research, read the appropriate section before selecting a response.

Materials & experimental systems

n/a	Involved in the study	
☐	☒	Antibodies
☐	☒	Eukaryotic cell lines
☒	☐	Palaeontology and archaeology
☐	☒	Animals and other organisms
☒	☐	Clinical data
☒	☐	Dual use research of concern
☒	☐	Plants

Methods

n/a	Involved in the study	
☒	☐	ChIP-seq
☐	☒	Flow cytometry
☒	☐	MRI-based neuroimaging

Antibodies

Antibodies used: We used commercially-available antibodies as per Materials and Methods; see "Confocal microscopy, Flow cytometry, and Immunoblotting".
The primary antibodies used in this study as follows:
rabbit anti-ZO-1 (61-7300; Thermo Scientific, 1:100 for immunofluorescence staining and 1:500 for immunoblotting)
mouse anti-ZO-1 (33-9100; Thermo Scientific, 1:100 for immunofluorescence staining and 1:1,000 for immunoblotting)
mouse anti-hCD47 (B6H12.2) (MA5-11895; Thermo Scientific, 1:100 for immunofluorescence staining, 1:500 for immunoblotting, and 1:50 for neutralization)
goat anti-mCD47 (AF1866; R&D Systems, 1:50 for immunofluorescence staining and 1:500 for immunoblotting)
rabbit anti-α-tubulin (EPR16772) (Acetyl K40, ab179484; Abcam, 1:100 for immunofluorescence staining)
rabbit anti-MUC5AC (E3O9I) (61193S, Cell Signaling Technology, 1:200 for immunofluorescence staining)
mouse monoclonal anti-p63 (D-9) (sc-25268, Santa Cruz Biotechnology, 1:100 for immunofluorescence staining)
rabbit anti-S. aureus (ab20920; Abcam, 1:100 for immunofluorescence staining)
rabbit polyclonal anti-Influenza A NP (PA5-32242, Thermo Scientific, 1:100 for immunofluorescence staining)
mouse anti-ICAM-1 (G-5) (sc-8439; Santa Cruz Biotechnology, 1:1,000 for immunoblotting)
rabbit anti-p65 (C22B4) (4764S; Cell Signaling Technology, 1:1,000 for immunoblotting)
rabbit anti-phospho-p65 (93H1) (Ser536, 3033S; Cell Signaling Technology, 1:1,000 for immunoblotting)
mouse anti-β-actin (C4) (sc-47778; Santa Cruz Biotechnology, 1:2,000 for immunoblotting)
mouse anti-GAPDH (6C5) (sc-32233; Santa Cruz Biotechnology, 1:2,000 for immunoblotting)
mouse IgG isotype control antibody (31903; Invitrogen, 1:5,000 for flow cytometry and 1:1,000 for neutralization)
normal goat IgG control antibody (AB-108-C; R&D Systems, 1:500 for neutralization)
mouse anti-Human IFN-Lambda Receptor 1 (MMHLR-1) (21885; PBL assay science, 1:50 for neutralization)
goat anti-Interferon alpha/beta receptor 1 (ab10739; abcam, 1:50 for neutralization)

The secondary antibodies used in this study as follows:
Alexa Fluor 488 donkey anti-mouse IgG (A21202; Molecular Probes, 1:1,000 for immunofluorescence staining)
Alexa Fluor 488 donkey anti-rabbit IgG (A21206; Molecular Probes, 1:1,000 for immunofluorescence staining)
Alexa Fluor 568 goat anti-mouse IgG (A11004; Molecular Probes, 1:1,000 for immunofluorescence staining)
Alexa Fluor 568 goat anti-rabbit IgG (A11011; Molecular Probes, 1:1,000 for immunofluorescence staining)
Alexa Fluor 647 donkey anti-goat IgG (A21447; Molecular Probes, 1:1,000 for immunofluorescence staining)
Peroxidase AffiniPure goat anti-rabbit IgG (H+L) (111-035-003; Jackson ImmunoResearch, 1:1,000 for immunoblotting)
goat anti-mouse IgG (H+L)-HRP (SA001; GenDEPOT, 1:1,000 for immunoblotting)
mouse anti-goat IgG-HRP (sc-2354; Santa Cruz Biotechnology, 1:500 for immunoblotting)
mouse anti-hCD47 APC-conjugated antibody (B6H12) (ab134485; Abcam, 1:200 for flow cytometry)

Validation: All antibodies used for immunofluorescence staining and flow cytometry are commercially available and have been validated technical data sheet as per manufacturer's website showing positive staining as opposed to the negative staining of isotype control.
All antibodies were titrated in our laboratory prior to their use and internally validated for use in this project.
Influenza NP (NucleoProtein) and S. aureus specificity was validated by comparing mock versus infected human bronchial epithelial cells (HBECs).
Human CD47 specificity was validated by comparing mock versus infected human nasal epithelial cells (HNECs) and human bronchial epithelial cells (HBECs) and using double staining by Cell markers (α-tubulin, MUC5AC).

Mouse CD47 specificity was validated by using Foxj1-Cre;tdTomato mice.
Junction protein (ZO-1) specificity was validated by comparing mock versus infected HNECs and HBECs.
Cell markers (α-tubulin, MUC5AC, p63) were validated by using fully-differentiated HNECs and HBECs.
Expression of CD47 using flow cytometry was validated by comparing IgG isotype control antibody treated cells versus hCD47 APC-conjugated antibody (B6H12) treated cells.

All antibodies used for immunoblotting are also commercially available and have been verified by many references provided on the website of the companies that sell antibodies (link below).
rabbit anti-ZO-1 (61-7300; Thermo Scientific, 1:500)
Validation Refs. from the manufacturer's datasheet: https://www.thermofisher.com/antibody/product/ZO-1-Antibody-Polyclonal/61-7300
mouse anti-ZO-1 (33-9100; Thermo Scientific, 1:1,000)
Validation Refs. from the manufacturer's datasheet: https://www.thermofisher.com/antibody/product/ZO-1-Antibody-clone-ZO1-1A12-Monoclonal/33-9100
mouse anti-hCD47 (B6H12.2) (MA5-11895; Thermo Scientific, 1:500)
Validation Refs. from the manufacturer's datasheet: https://www.thermofisher.com/antibody/product/CD47-Antibody-clone-B6H12-2-Monoclonal/MA5-11895
goat anti-mCD47 (AF1866; R&D Systems, 1:500)
Validation Refs. from the manufacturer's datasheet: https://www.rndsystems.com/products/mouse-rat-cd47-n-terminal-igv-like-extracellular-domain-antibody_af1866#product-datasheets
mouse anti-ICAM-1 (G-5) (sc-8439; Santa Cruz Biotechnology, 1:1,000)
Validation Refs. from the manufacturer's datasheet: https://www.scbt.com/ko/p/icam-1-antibody-g-5
rabbit anti-p65 (C22B4) (4764S; Cell Signaling Technology, 1:1,000)
Validation Refs. from the manufacturer's datasheet: https://www.cellsignal.com/products/primary-antibodies/nf-kb-p65-c22b4-rabbit-mab/4764
rabbit anti-phospho-p65 (93H1) (Ser536, 3033S; Cell Signaling Technology, 1:1,000)
Validation Refs. from the manufacturer's datasheet: https://www.cellsignal.com/products/primary-antibodies/phospho-nf-kb-p65-ser536-93h1-rabbit-mab/3033
mouse anti-β-actin (C4) (sc-47778; Santa Cruz Biotechnology, 1:2,000)
Validation Refs. from the manufacturer's datasheet: https://www.scbt.com/ko/p/beta-actin-antibody-c4
mouse anti-GAPDH (6C5) (sc-32233; Santa Cruz Biotechnology, 1:2,000)
Validation Refs. from the manufacturer's datasheet: https://www.scbt.com/ko/p/gapdh-antibody-6c5

Eukaryotic cell lines

Policy information about cell lines and Sex and Gender in Research

Cell line source(s)	Human bronchial epithelial cells (HBECs) were purchased from Lonza (CC-2540S). HBECs used in this study were obtained from four non-asthmatic adult donors: a 43-year-old male (Hispanic), a 52-year-old male (Hispanic), a 66-year-old male (Hispanic), and a 48-year-old female (Caucasian).<br>Madin-Darby canine kidney (MDCK) cells (CCL-34) and A549 cells (CCL-185) were obtained from American Type Culture Collection (ATCC).
Authentication	We did not use cell lines that require new authentication.
Mycoplasma contamination	The manufacturer confirms that there is no mycoplasma contamination in their product.
Commonly misidentified lines (See ICLAC register)	We did not use any misidentified cell lines in this study.

Animals and other research organisms

Policy information about studies involving animals; ARRIVE guidelines recommended for reporting animal research, and Sex and Gender in Research

Laboratory animals	All used mice in this study were housed under specific pathogen-free conditions and used at 6–8 weeks of age.<br>C57BL/6 male mice purchased from Orient Bio (Gyeonggi, Korea) were used in the experiments.<br>C57BL/6J background CD47 floxed (Cd47f/f) mice were purchased from the Mouse Biology Program at UC Davis, CA, USA (Cd47tm1c(KOMP)Mbp/Mmucd, 046999-UCD).<br>Rosa26-tdTomato reporter mice were kindly provided by Dr. Jinwoong Bok (Yonsei University College of Medicine, Seoul, Republic of Korea). Foxj1Cre transgenic and LysMCre knock-in/knock-out mice were kindly provided by Dr. Michael J. Holtzman (Washington University School of Medicine, St. Louis, MO) and Dr. Hyoung-Pyo Kim (Yonsei University College of Medicine, Seoul, Republic of Korea), respectively. Foxj1Cre;Cd47f/f (Cd47Foxj1), LysMCre;Cd47f/f (Cd47LysM), and Cd47f/f mice were bred in-house at Yonsei University College of Medicine in Seoul, Republic of Korea.<br>Mice were housed in the specific pathogen-free (SPF) animal facilities under a 12 h light-dark cycle at 20 ± 2 °C, a humidity of 50 ± 5%, ventilation of 10-15/h, the light of 150-300 Lux, noise of less than 60 dB and maintained on normal chow diet.
Wild animals	We did not use any wild animals in this study.
Reporting on sex	Only male animals were used in this study. Because phenotype of super-infection is not expected to be different between males and females.
Field-collected samples	We did not use any field-collected samples in this study.

Ethics oversight | All animal work was approved by the Institutional Animal Care and Use Committee (IACUC) at Yonsei University College of Medicine (protocol number 2022-0257), according to guidelines outlined by the Association for Assessment and Accreditation of Laboratory Animal Care (AAALAC) International (facility number 001071).

Note that full information on the approval of the study protocol must also be provided in the manuscript.

Plants

Seed stocks | We did not use any seed stocks in this study.

Novel plant genotypes | We did not use any novel plant genotypes in this study.

Authentication | N/A

Flow Cytometry

Plots

Confirm that:

☒ The axis labels state the marker and fluorochrome used (e.g. CD4-FITC).

☒ The axis scales are clearly visible. Include numbers along axes only for bottom left plot of group (a 'group' is an analysis of identical markers).

☒ All plots are contour plots with outliers or pseudocolor plots.

☒ A numerical value for number of cells or percentage (with statistics) is provided.

Methodology

Sample preparation | Human nasal epithelial cells (HNECs) were detached from transwell plate and seperated using a mixture of non-enzymatic cell dissociation solution (Signma Aldrich) and trypsin-EDTA in a 1:1 ratio. Cells were centrifuged and resuspended in PBS containing 2% fetal bovine serum (FBS) and filtered through a 40µm stainer.
Cells were incubated with 1µg of mouse IgG isotype control antibody (Invitrogen) or mouse anti-hCD47 APC-conjugated antibody (Abcam) for 20 min on ice and then washed. The washed pellet was resuspended in PBS containing 2% fetal bovine serum (FBS) and then the samples were analyzed using flow cytometry. Nonviable cells were excluded from further analyses.

Instrument | The stained cells were analyzed using an BD FACSLyric™ (BD Biosciences).

Software | Data were acquired with BD FACSuite v1.3 (BD Biosciences) and analyzed with FlowJo™v.10.6.2 software (BD Biosciences).

Cell population abundance | Epithelial cells were stained and run without sorting or enrichment. Cell population abundance was not analyzed.

Gating strategy | For the experiments, single cells were gated on a forward scatter height vs. forward scatter area plot. Epithelial cells were gated on forward vs. side scatter plot based on their size and granularity. To exclude dead cells, 7-AAD+ cells were gated out. Subsequently, CD47 expression was analyzed.

☒ Tick this box to confirm that a figure exemplifying the gating strategy is provided in the Supplementary Information.
