# Supplementary material for: Airway epithelial CD47 plays a critical role in inducing influenza virus-mediated bacterial super-infection
Source: Nat Commun. 2024 Apr 30;15:3666. doi: 10.1038/s41467-024-47963-5 (PMC11063069; doi:10.1038/s41467-024-47963-5)

Fig. 1a

Western blot bands (HBECs)

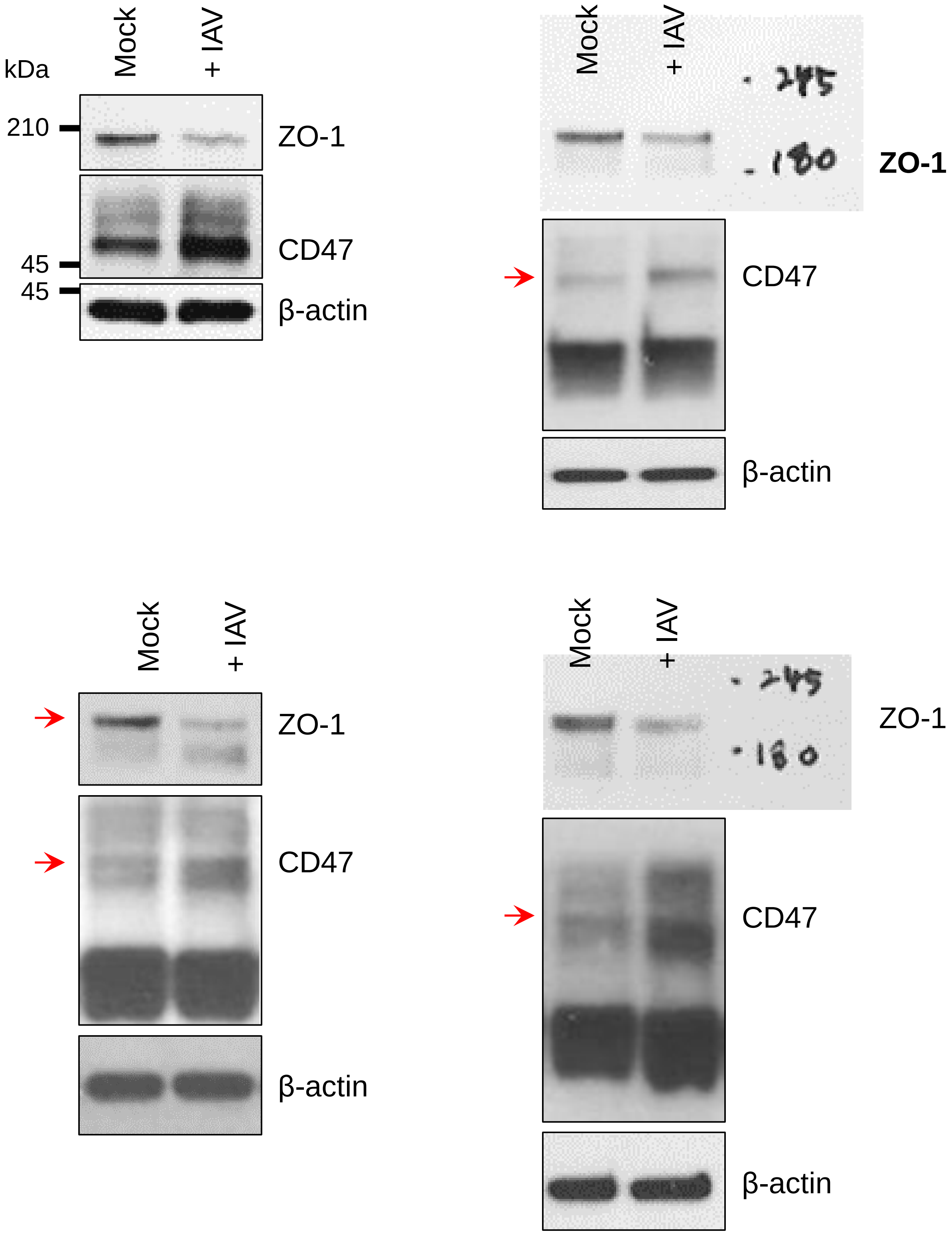

Fig. 1b

Whole-mount images (HBECs)

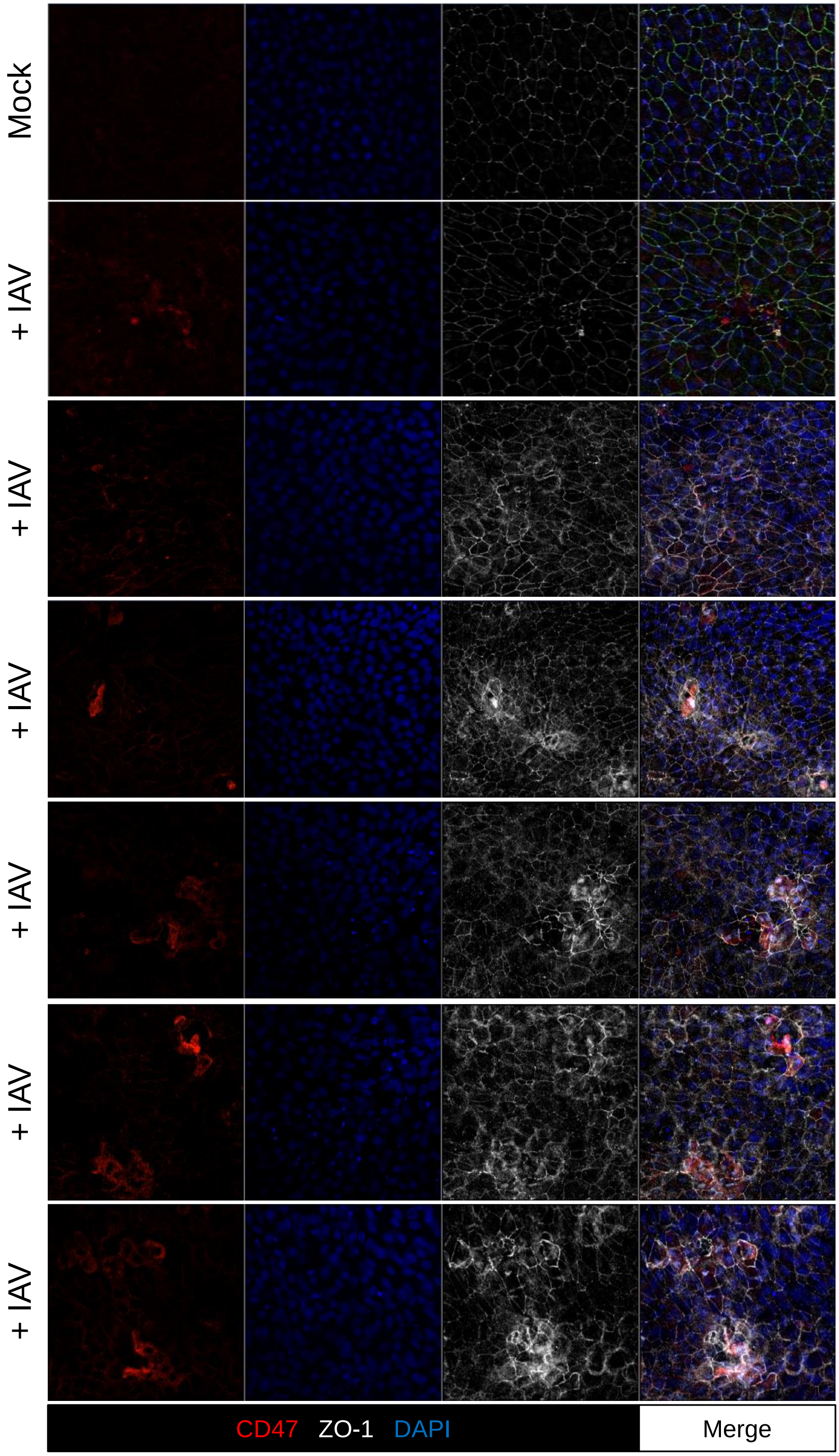

Fig. 1d

Western blot bands (HBECs)

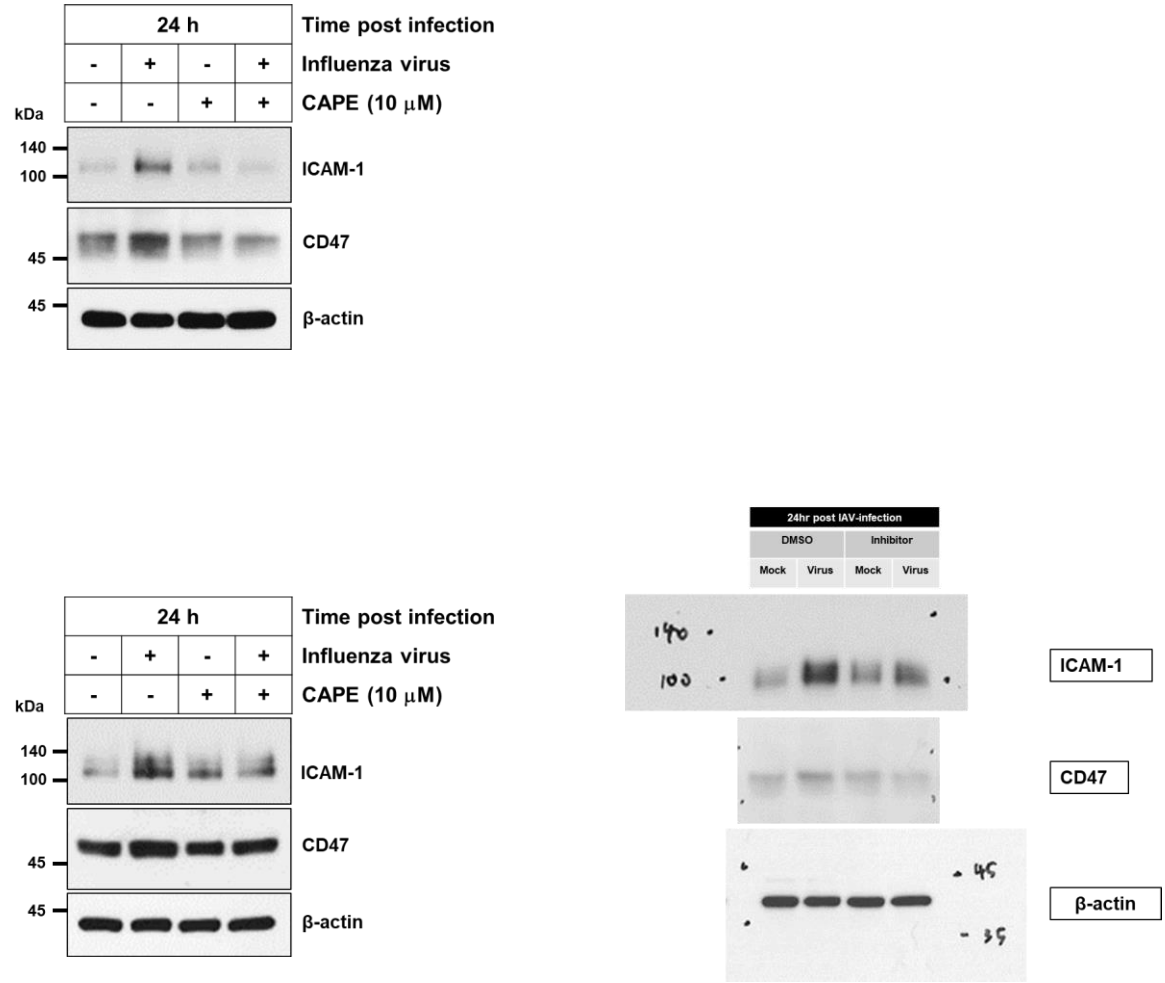

Fig. 1e

Whole-mount images (HBECs)

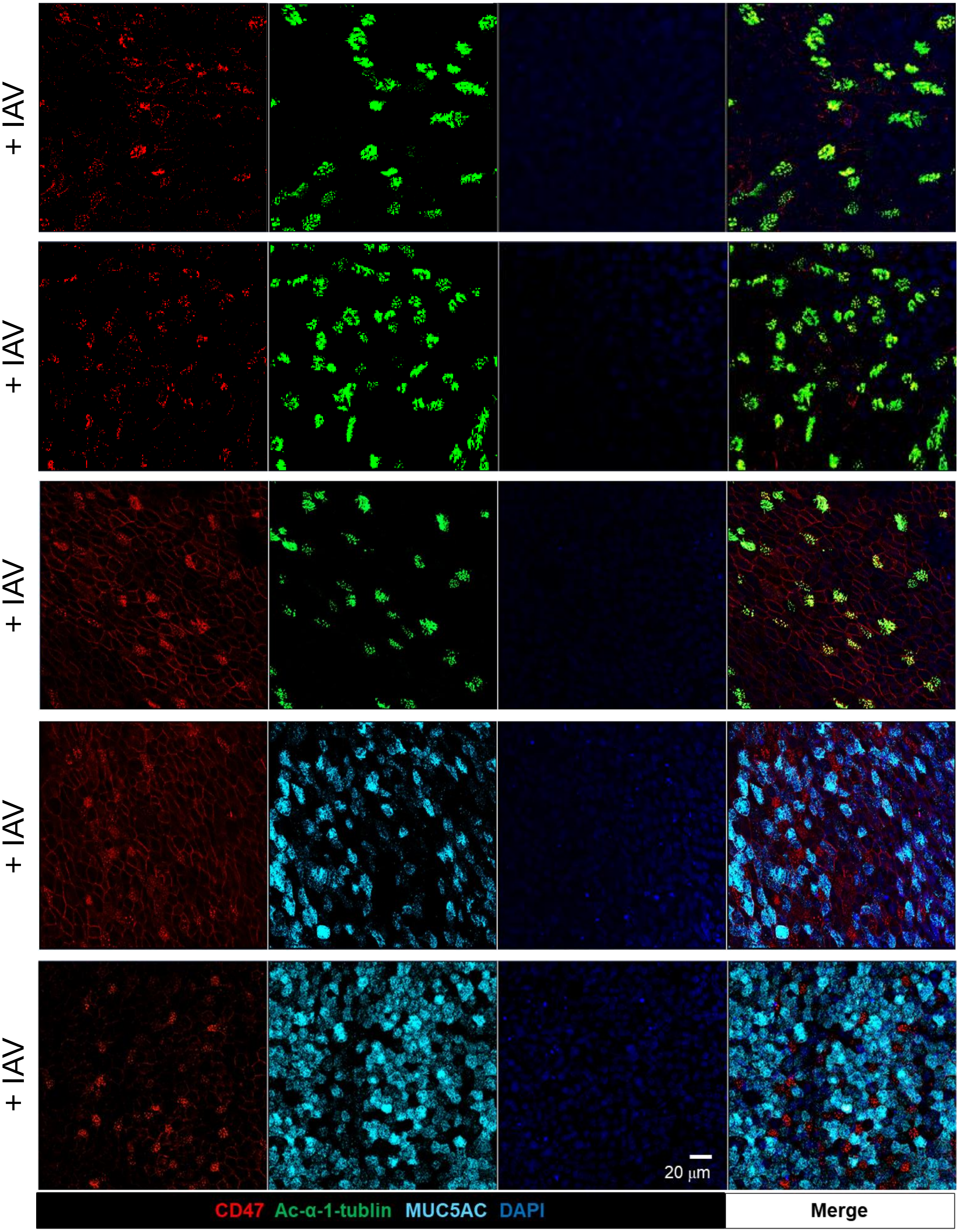

Fig. 2b

Western blot bands (HBECs)

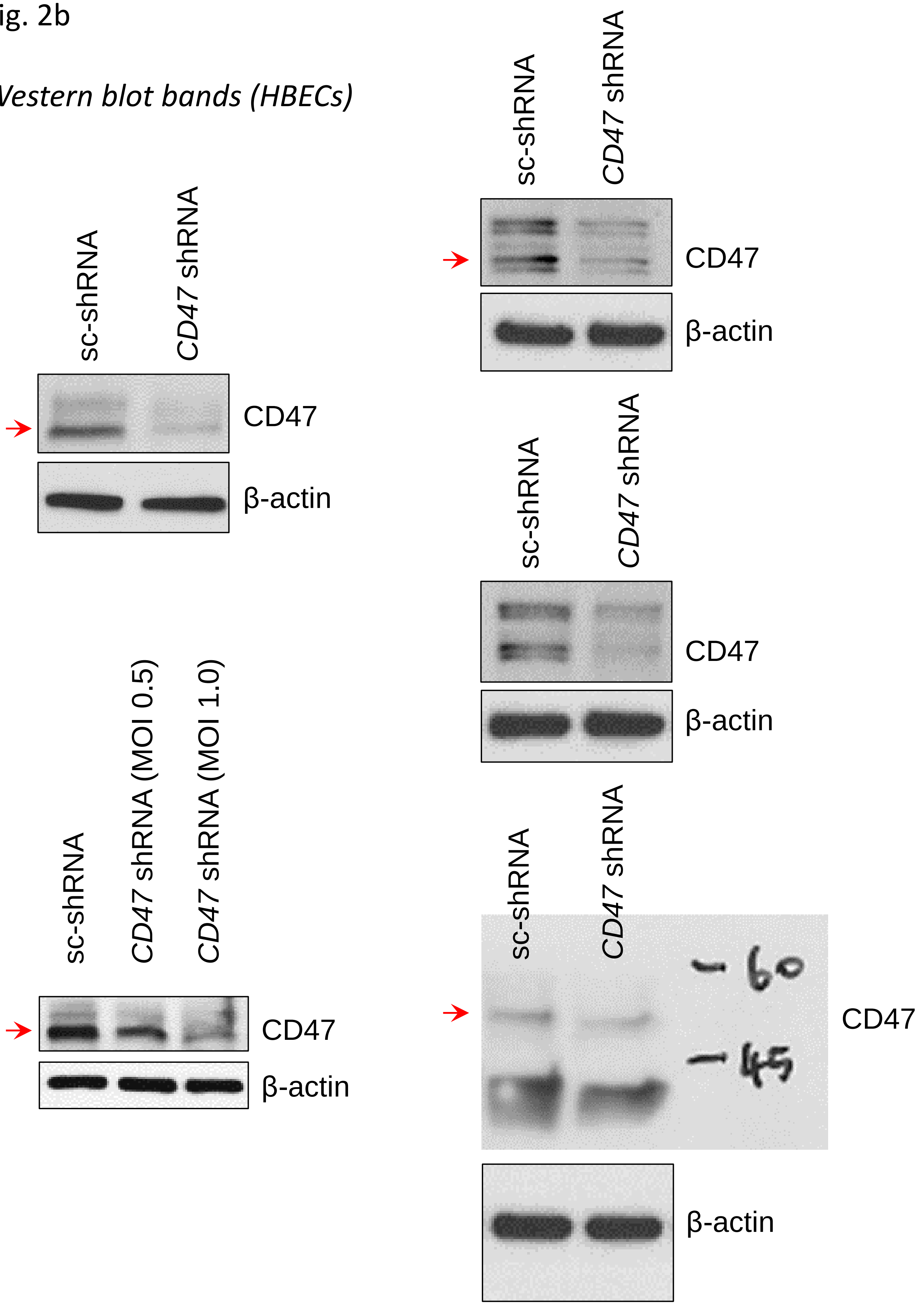

Fig. 2e

Microscopic images (HBECs)

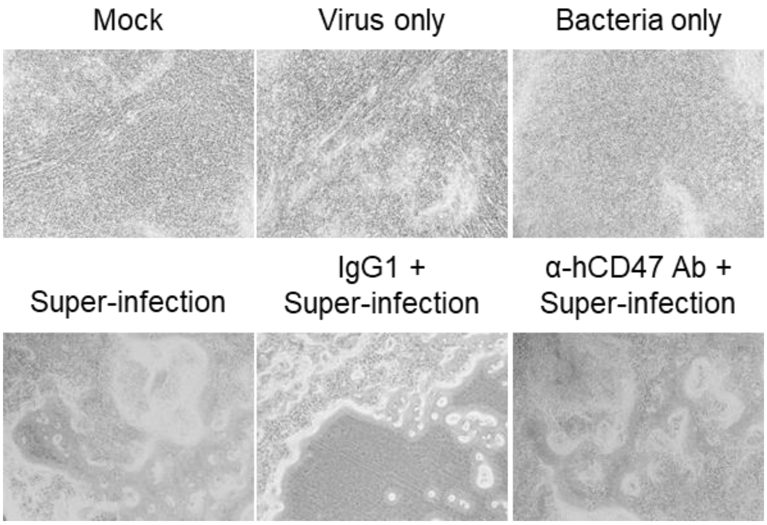

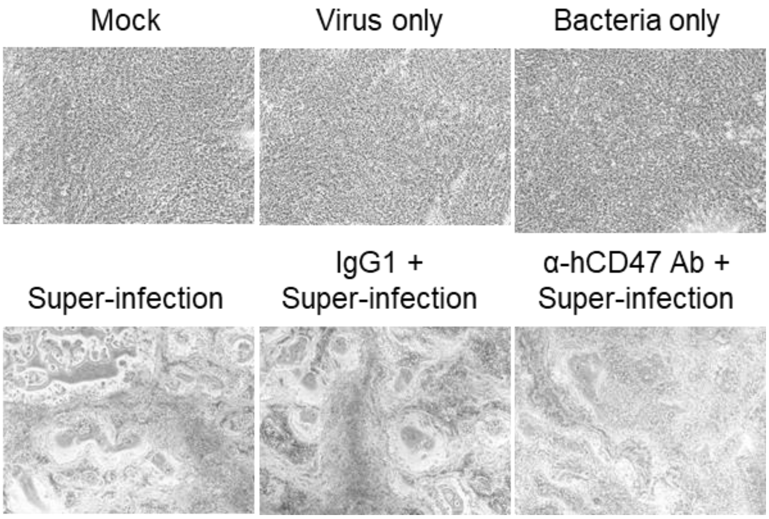

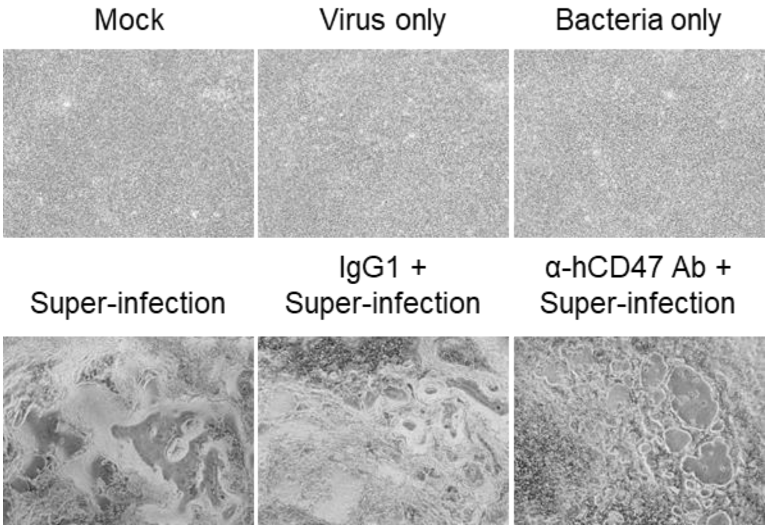

Fig. 3c

Whole-mount images (HBECs)

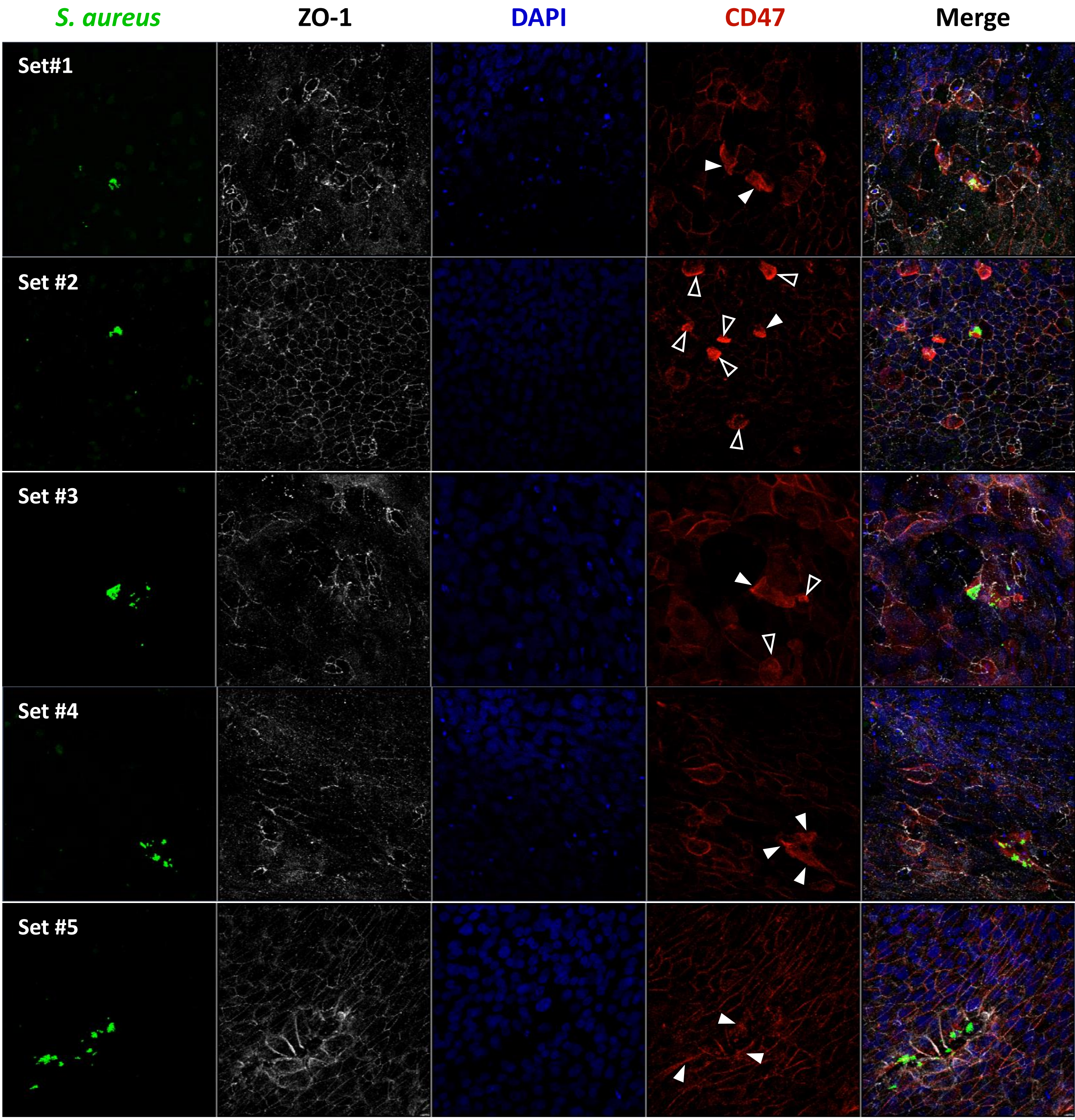

Fig. 3h

In vitro pull-down assay

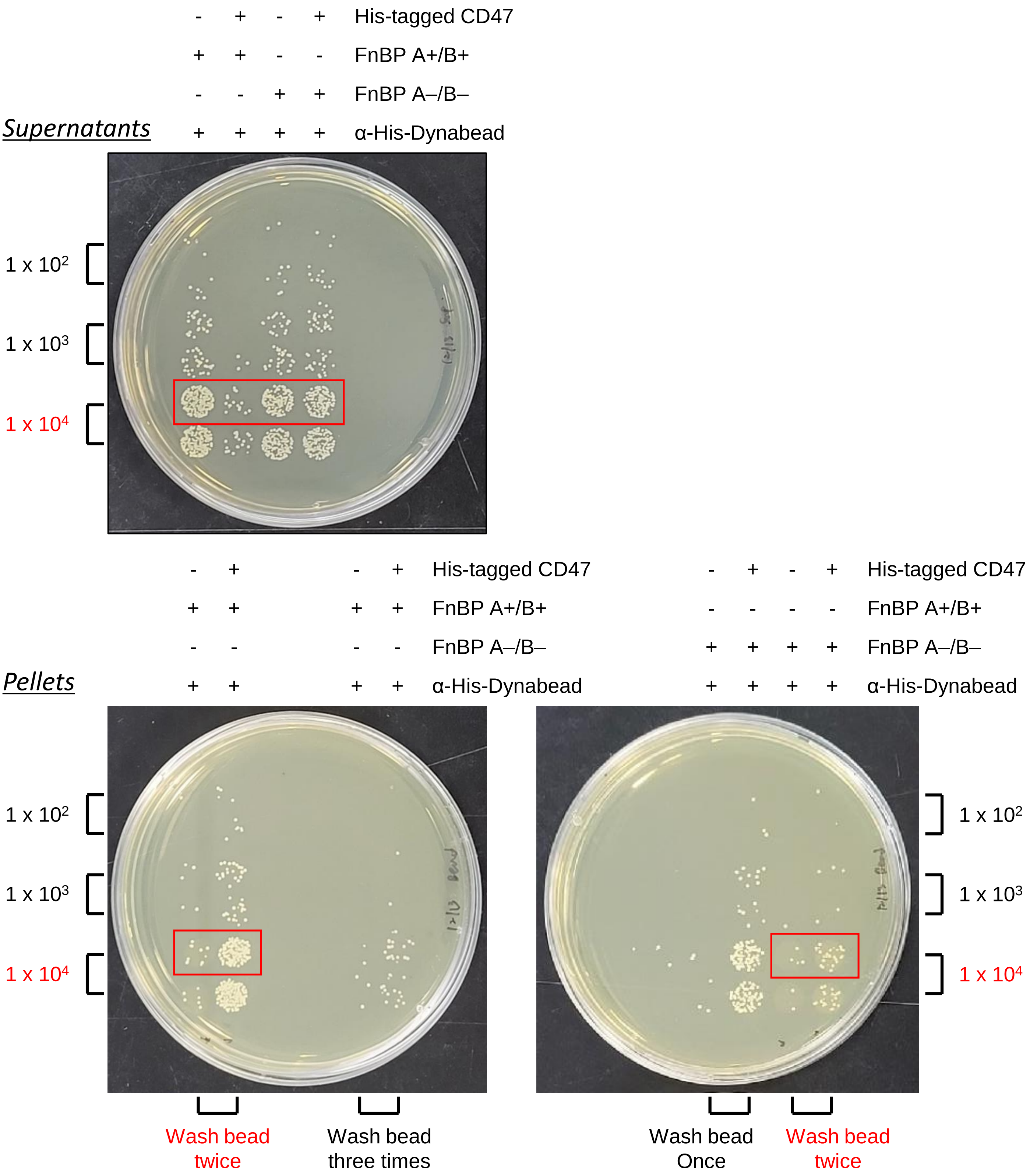

Fig. 4c

H&E staining

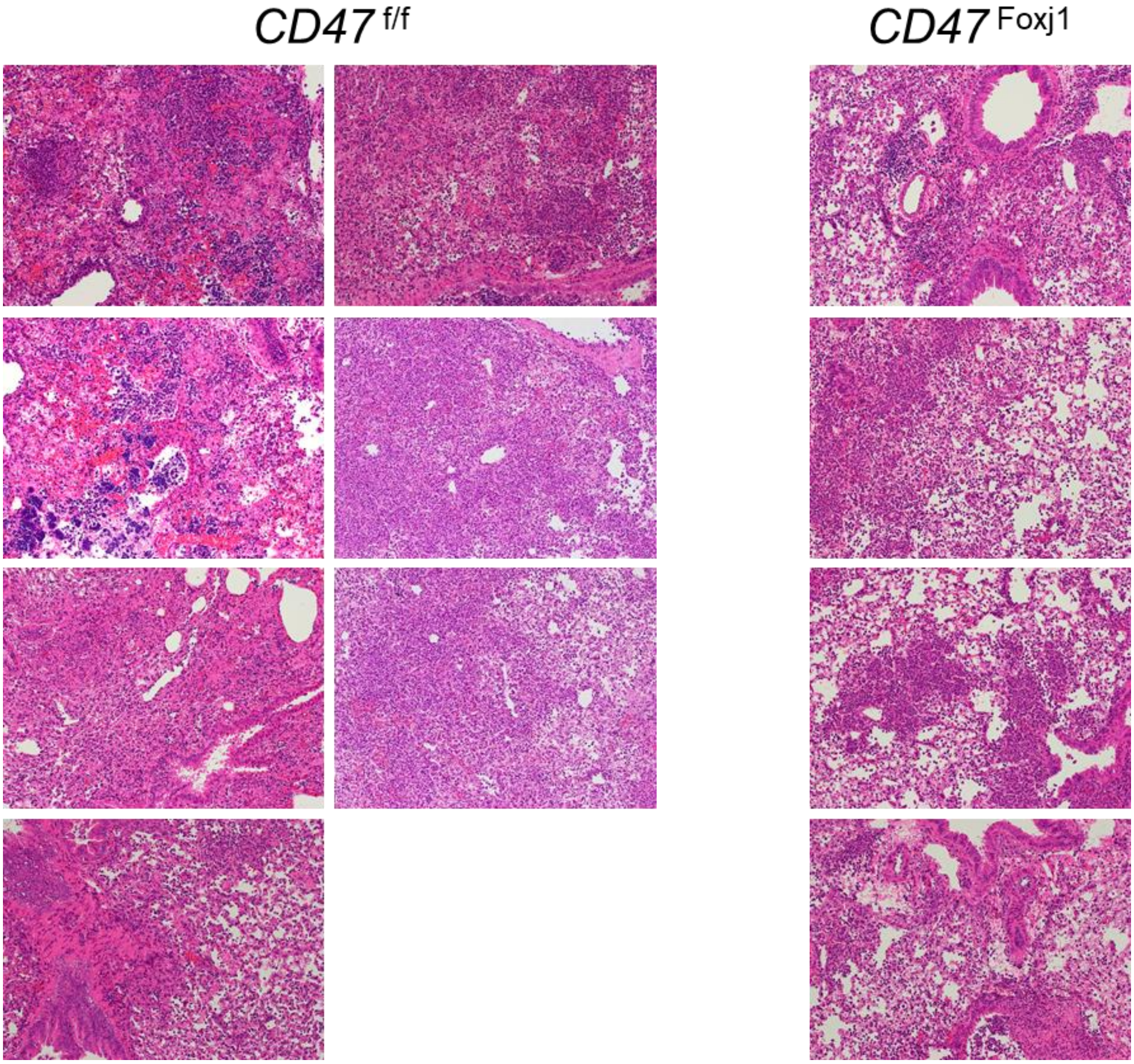

Fig. 4k

H&E staining

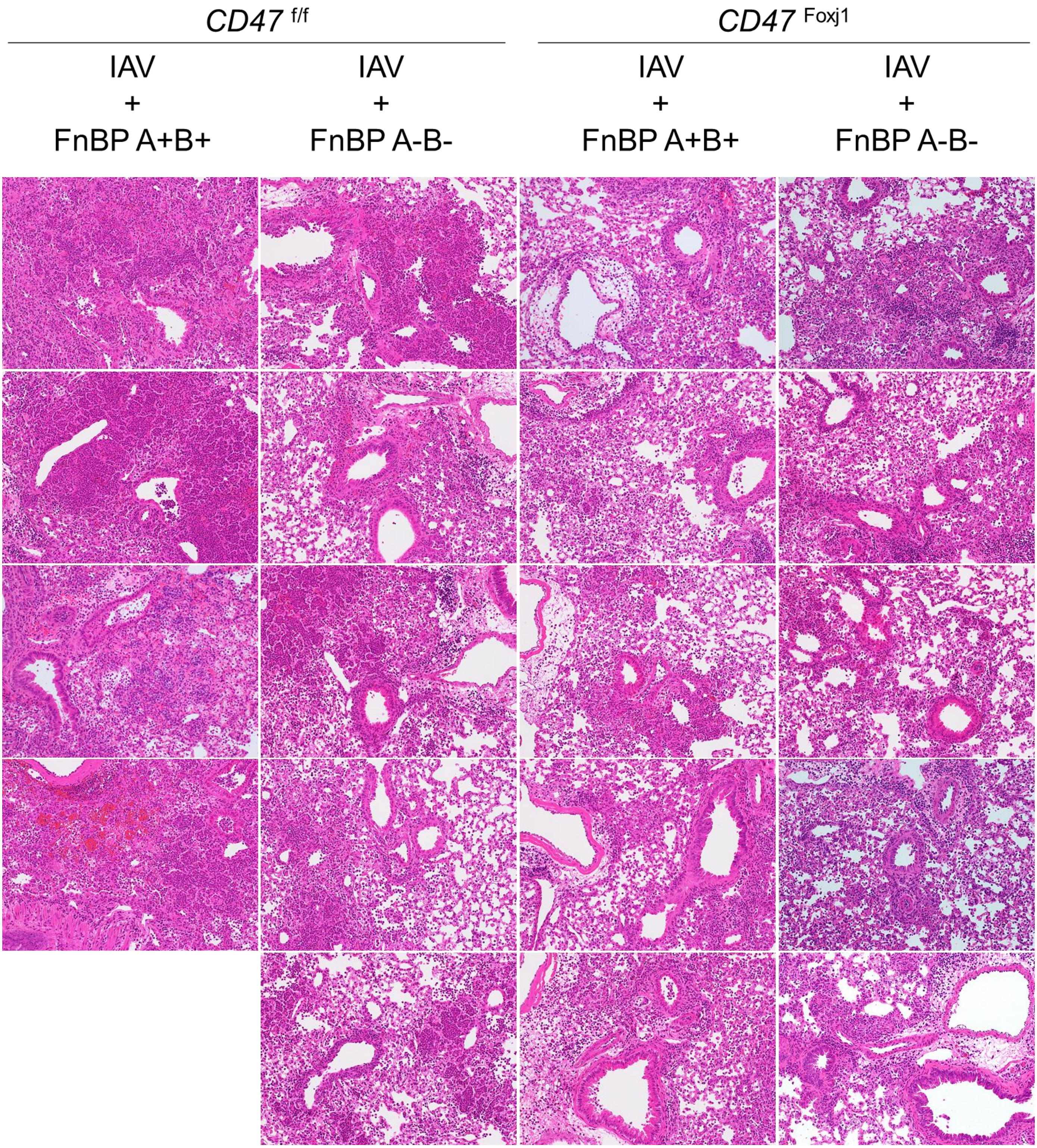

Fig. 4l

H&E staining

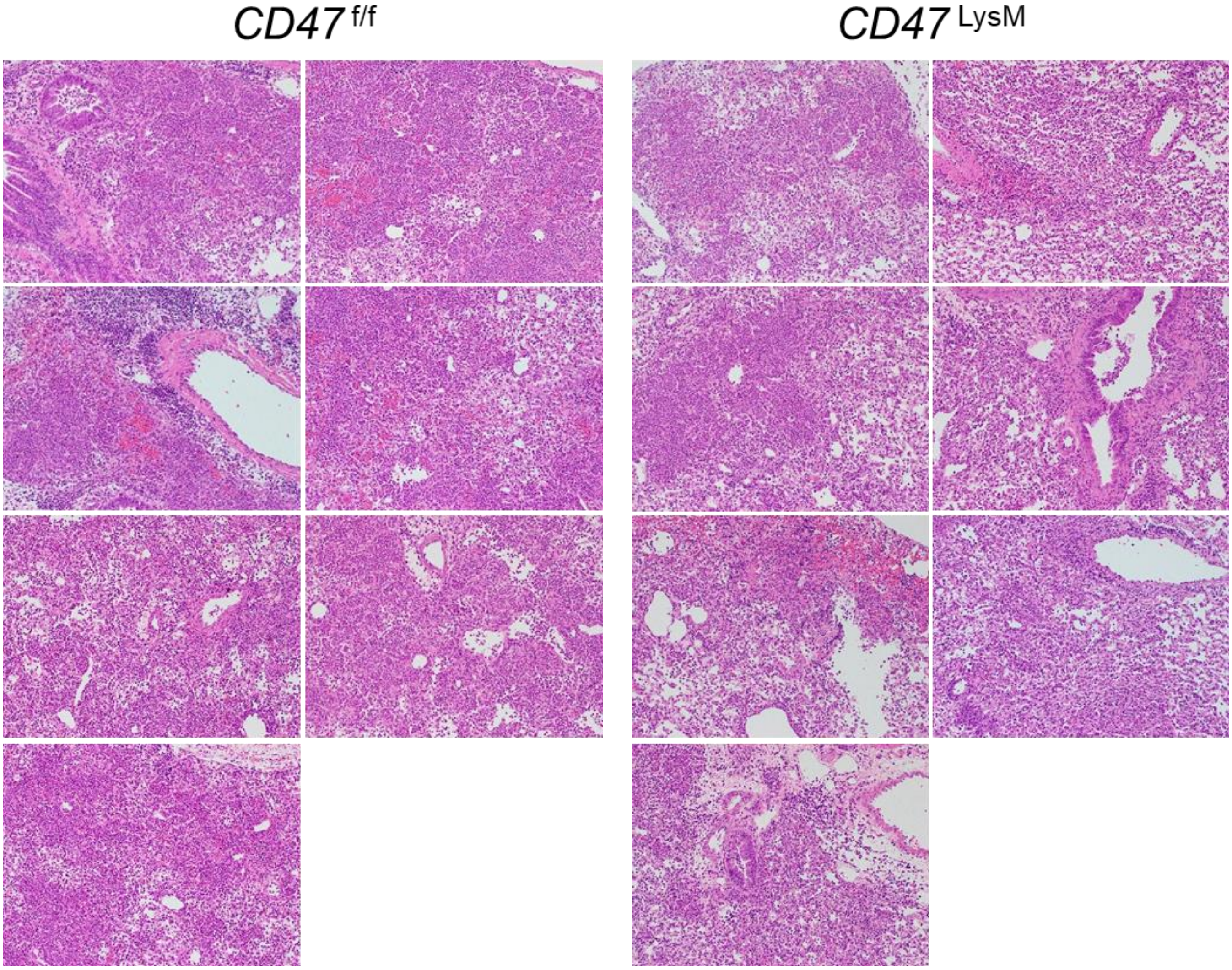

Fig. 5c

H&E staining

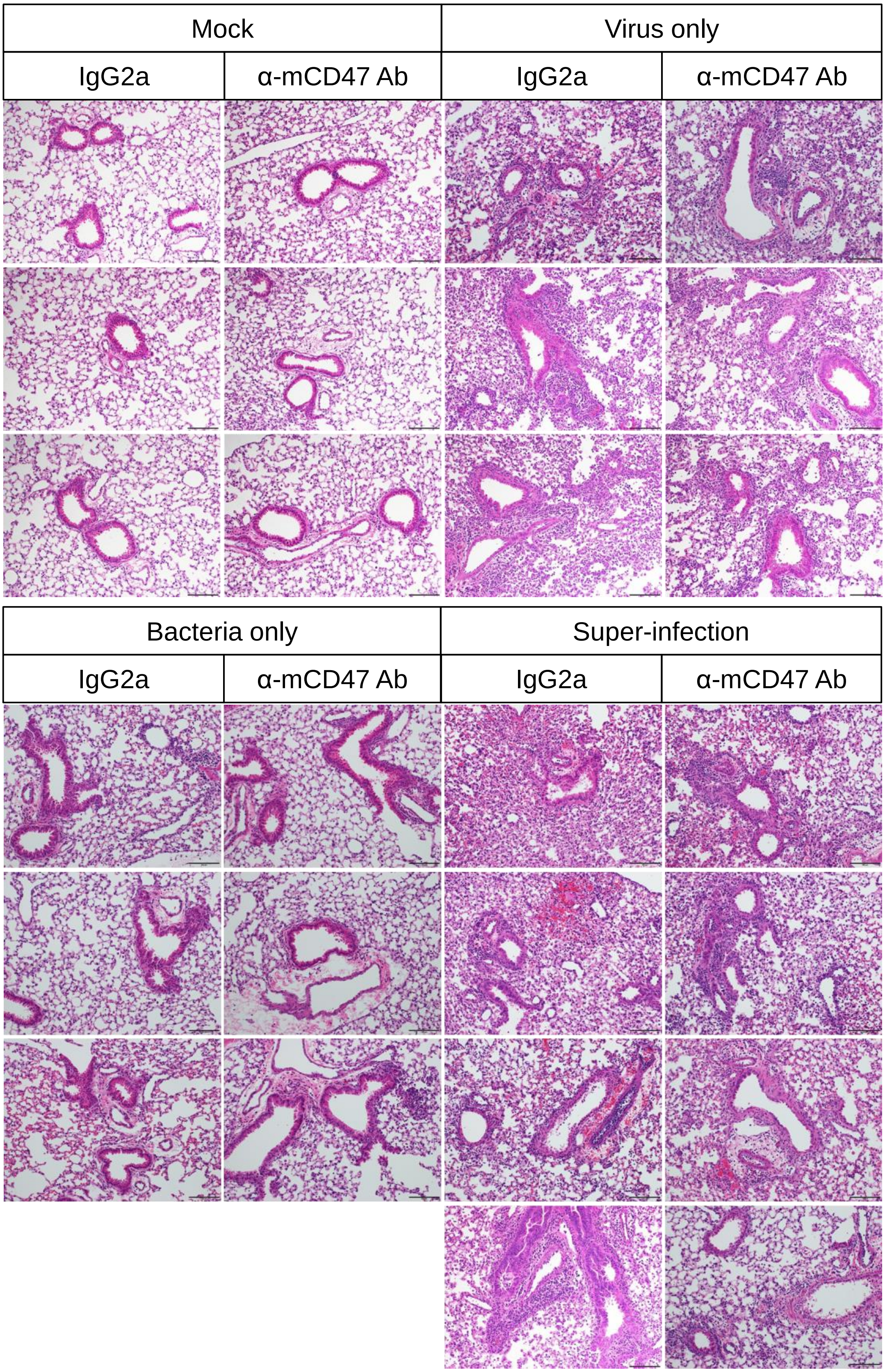

Fig. S2a

Western blot bands (HNECs)

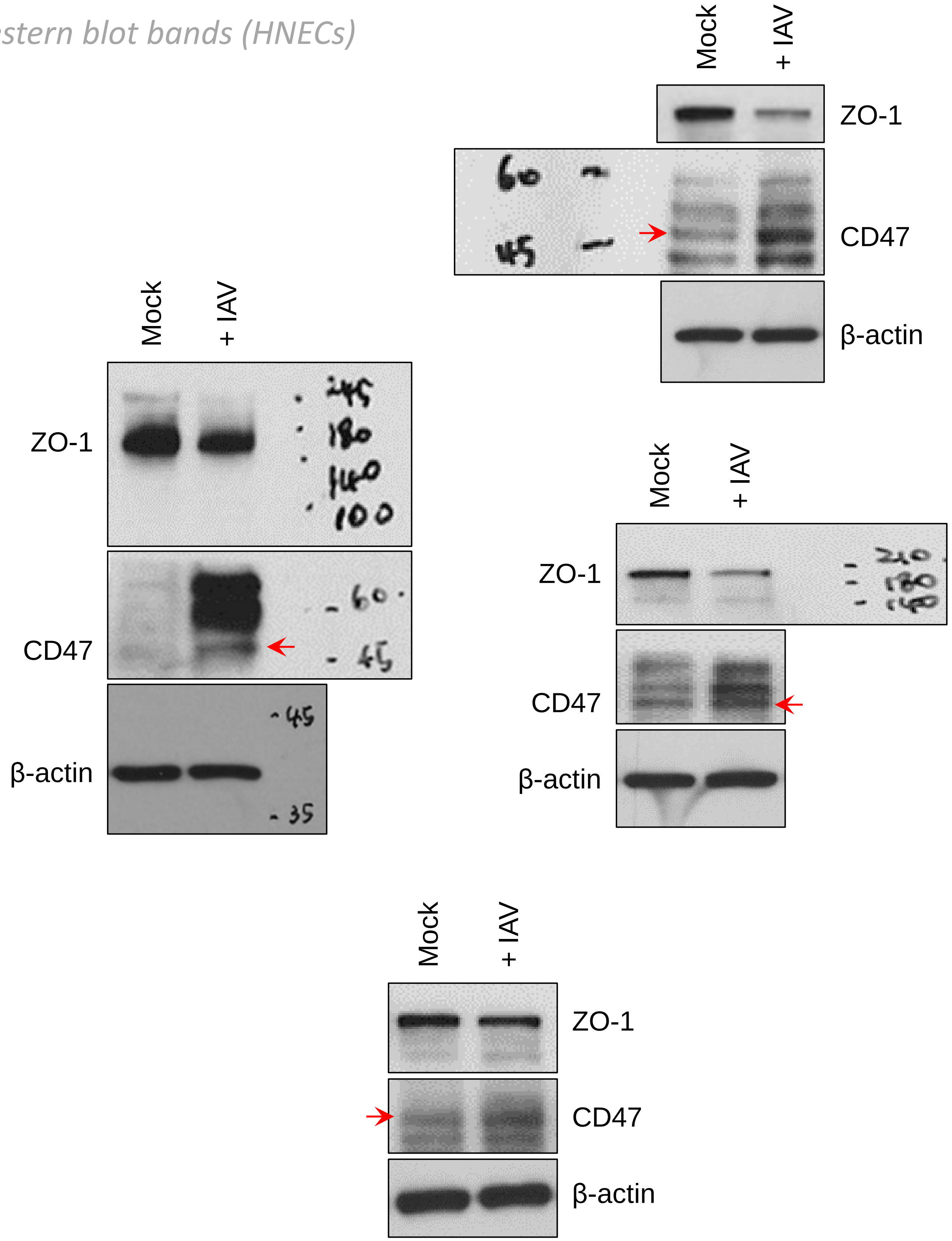

Fig. S2b

Whole-mount images (HNECs)

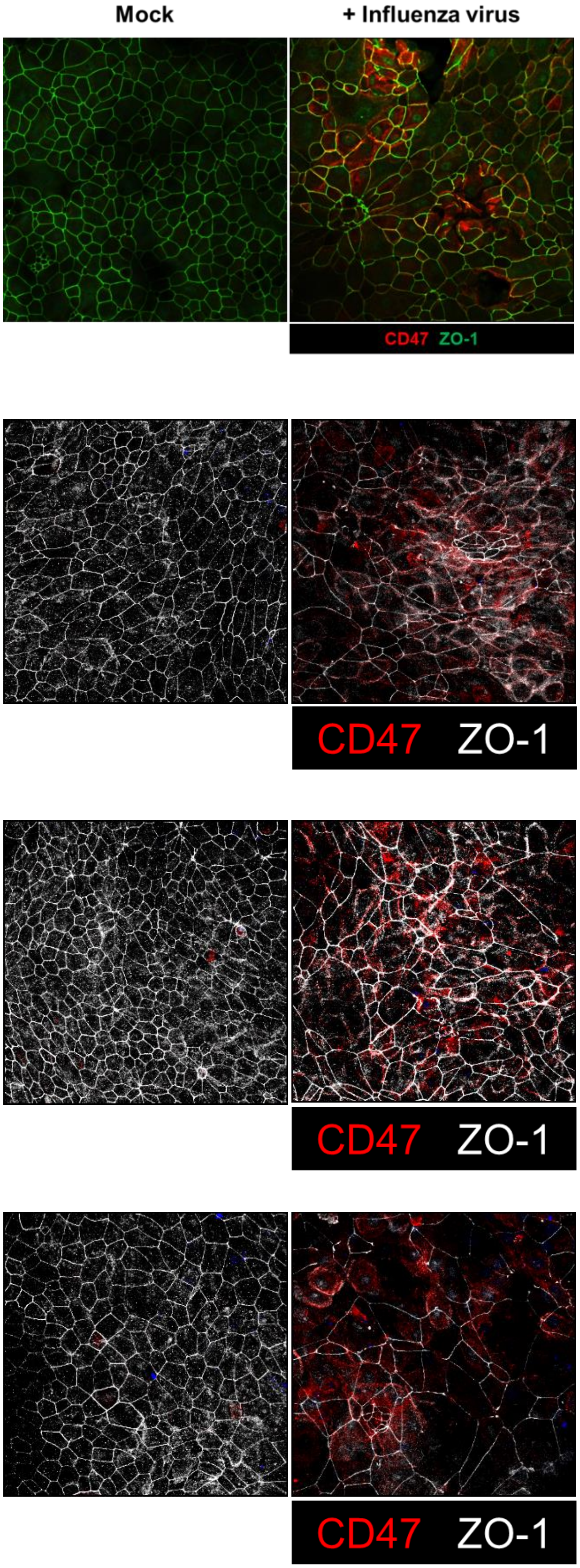

Fig. S2d

Whole-mount images (HNECs)

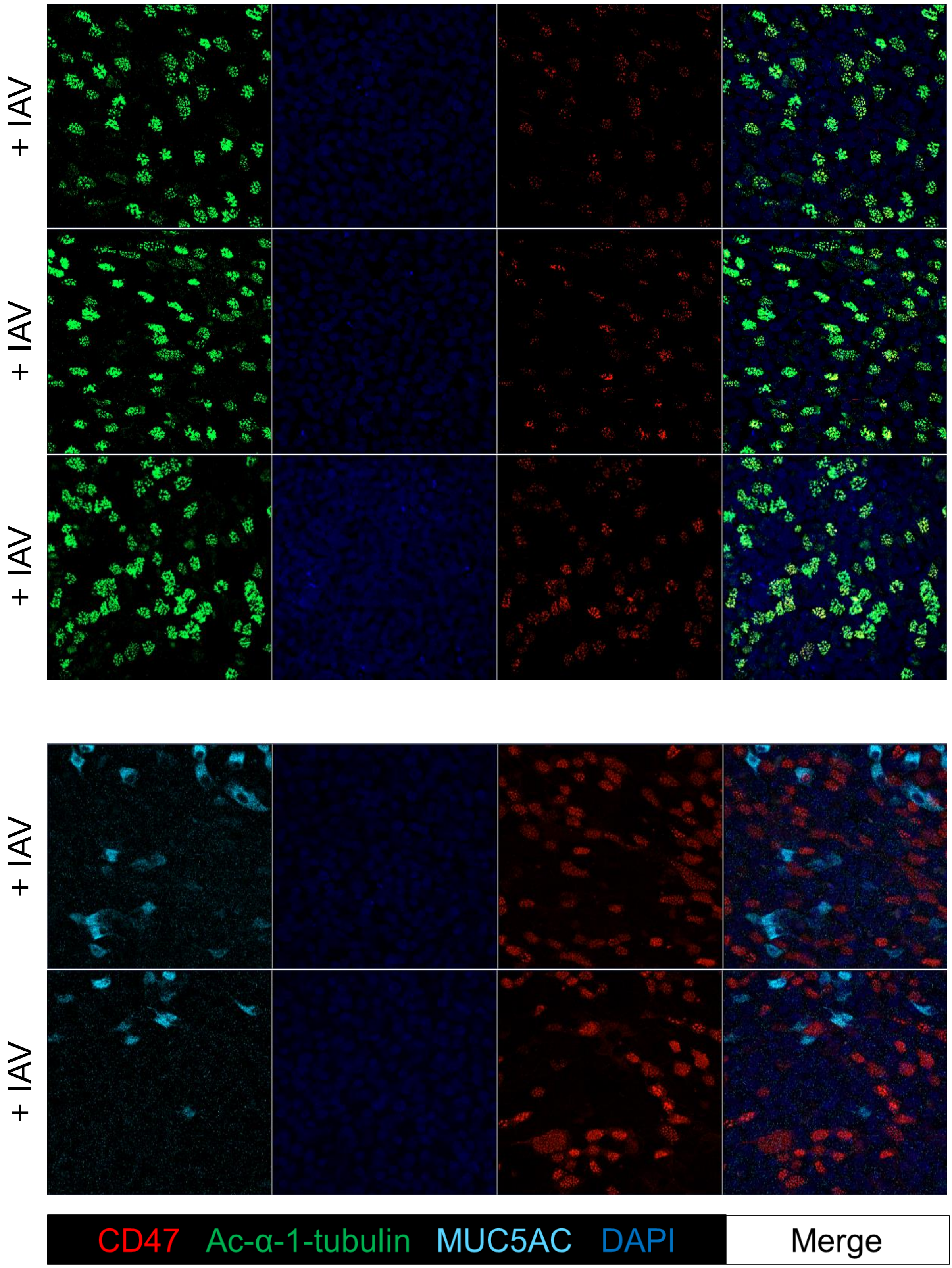

Fig. S3a

Western blot bands (HBECs)

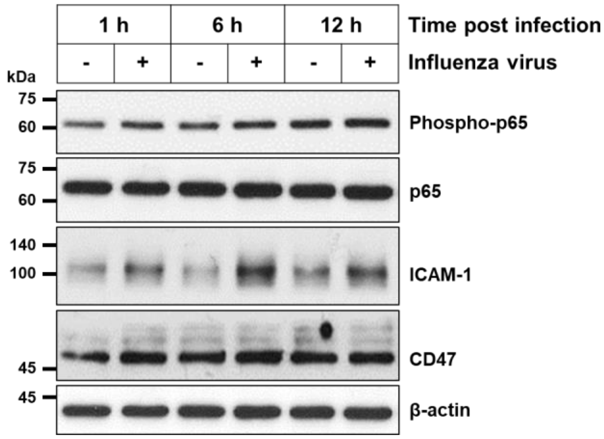

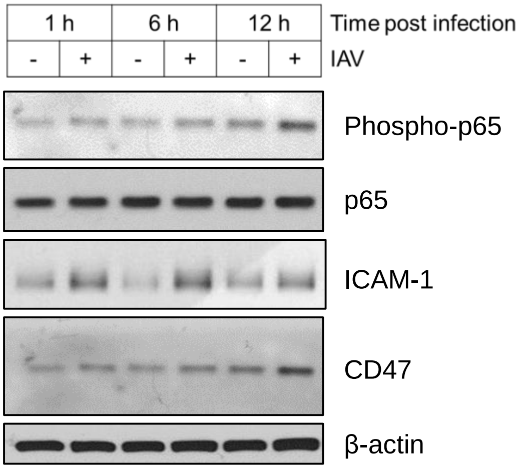

Fig. S3b

Western blot bands (HBECs)

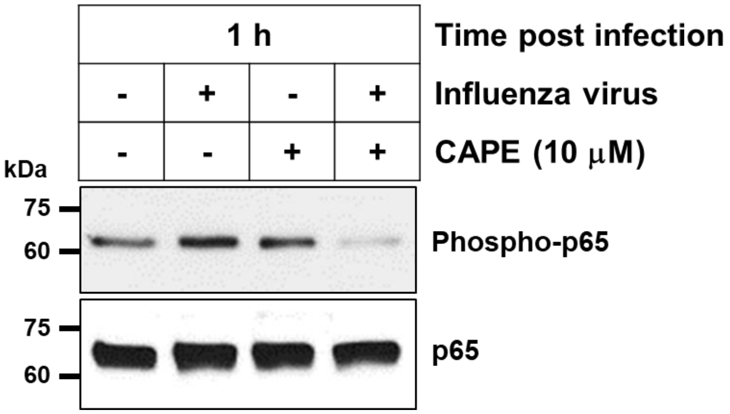

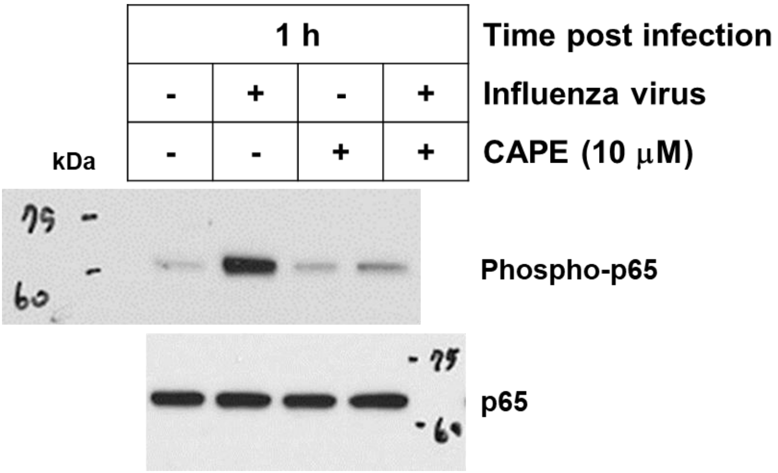

Fig. S3g

Whole-mount images (HBECs)

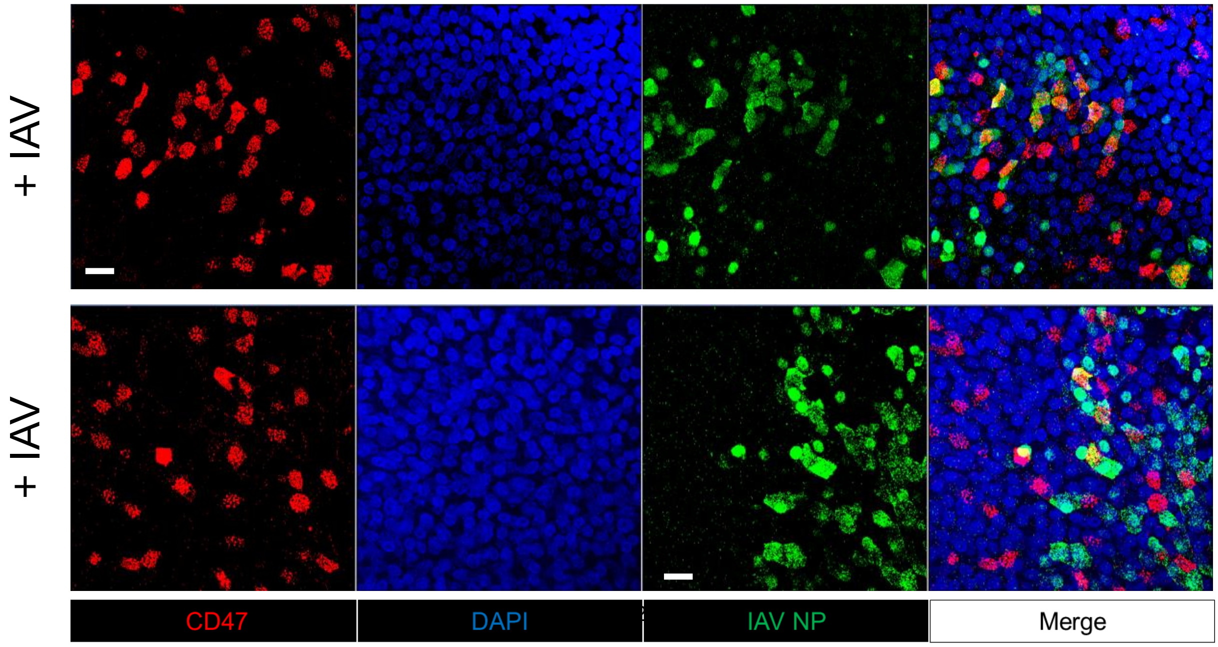

Fig. S4d

Microscopic images (HNECs)

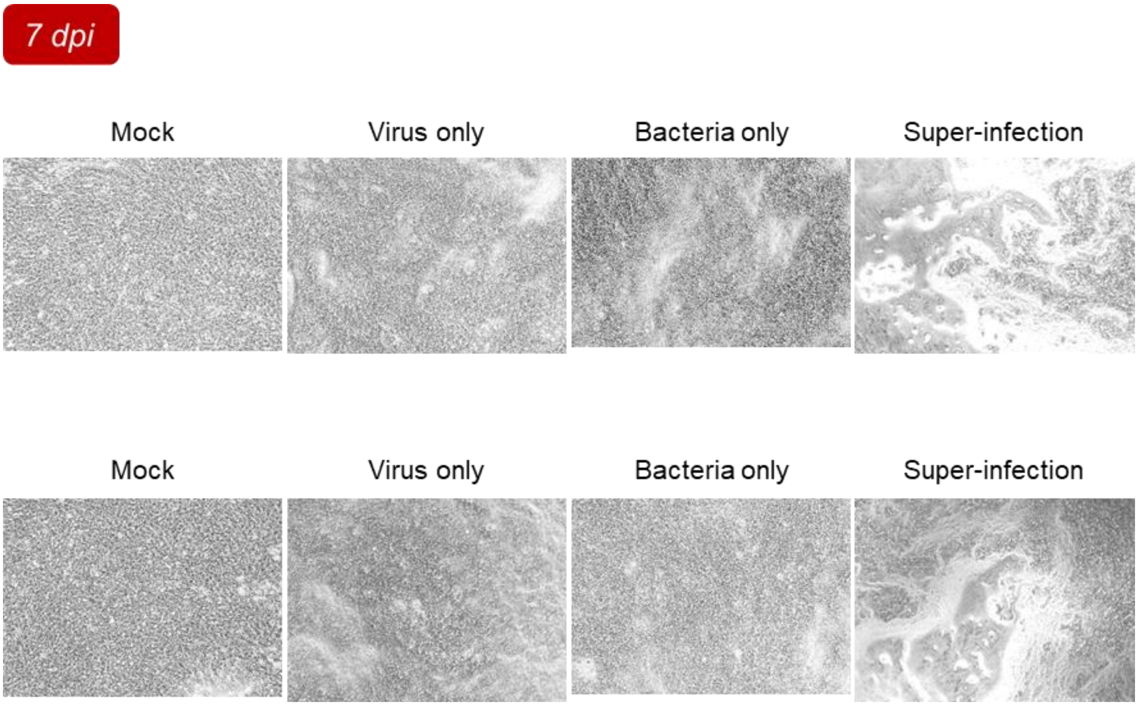

Fig. S4h

Microscopic images (HBECs)

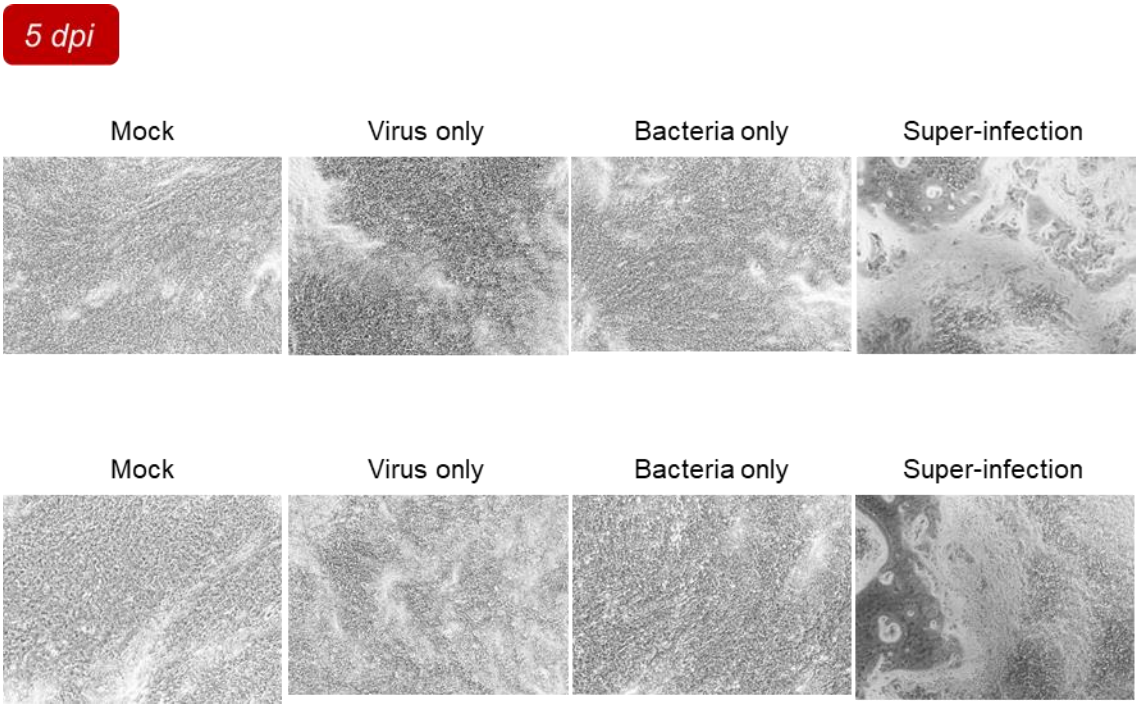

Fig. S5b

Western blot bands (HNECs)

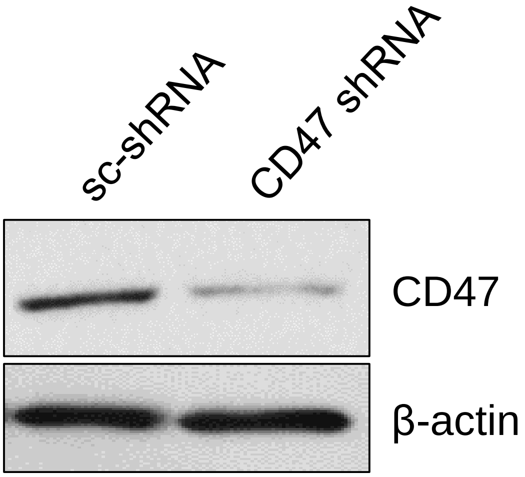

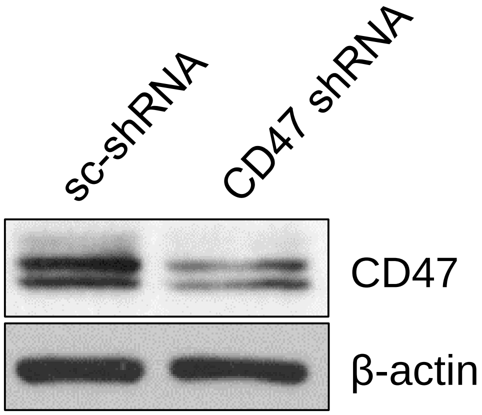

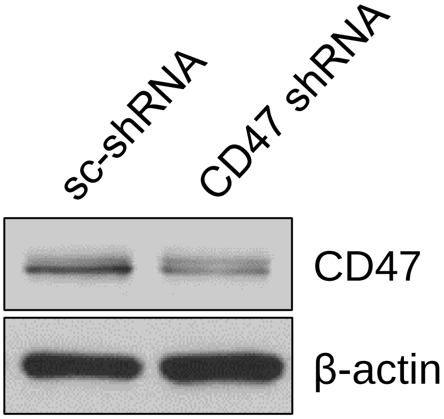

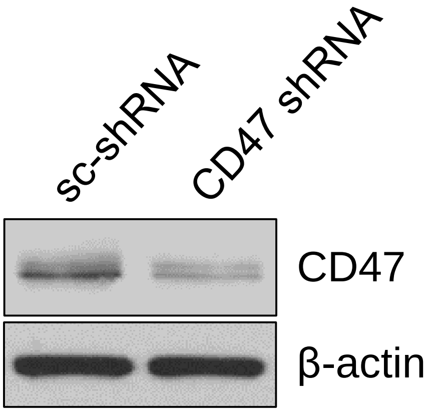

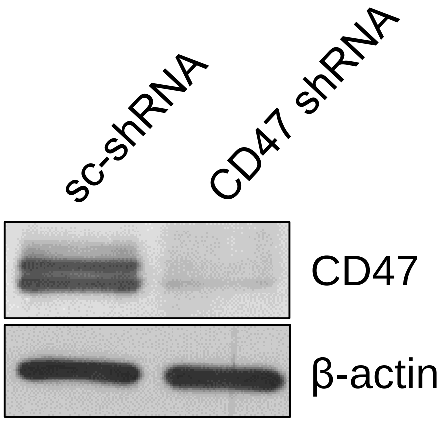

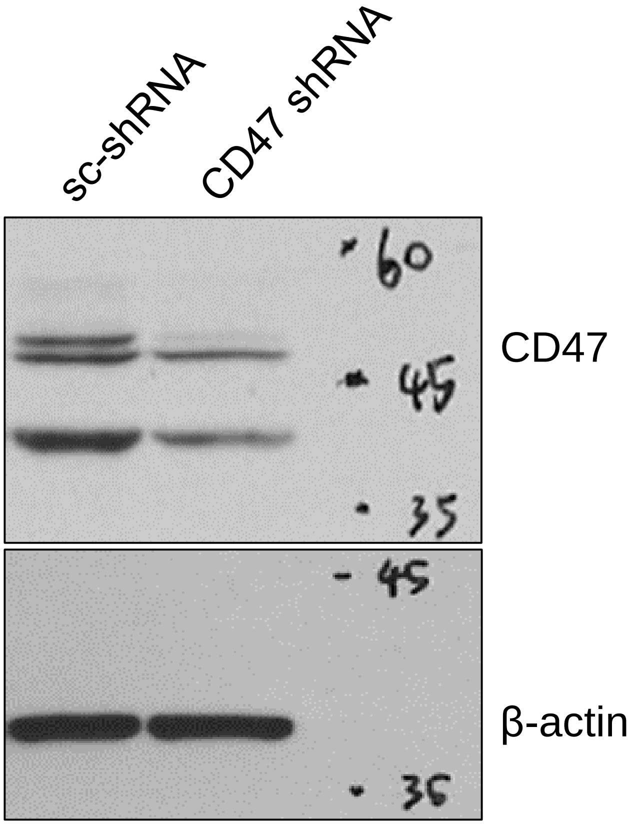

Fig. S5g

Microscopic images

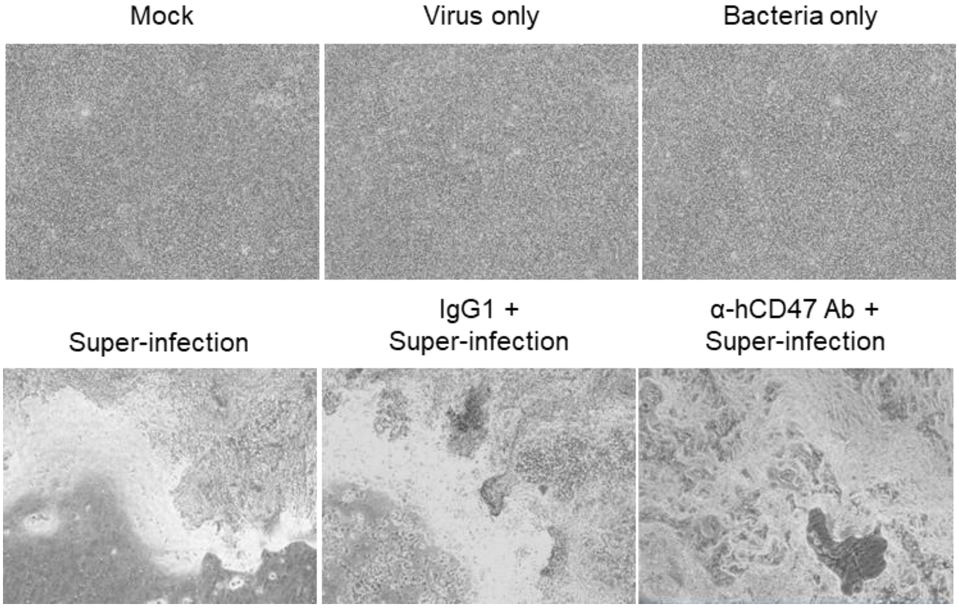

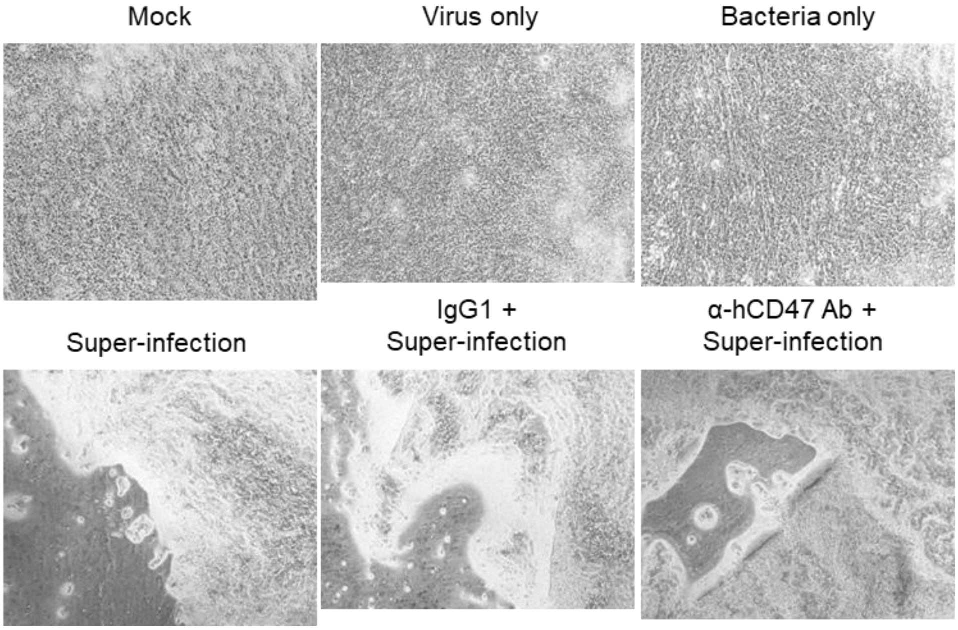

Fig. S8c

Western blot bands (in vivo)

1 set post IAV infection day lung sample western blot data (mock, *n = 15* / IAV infected, *n = 15*)

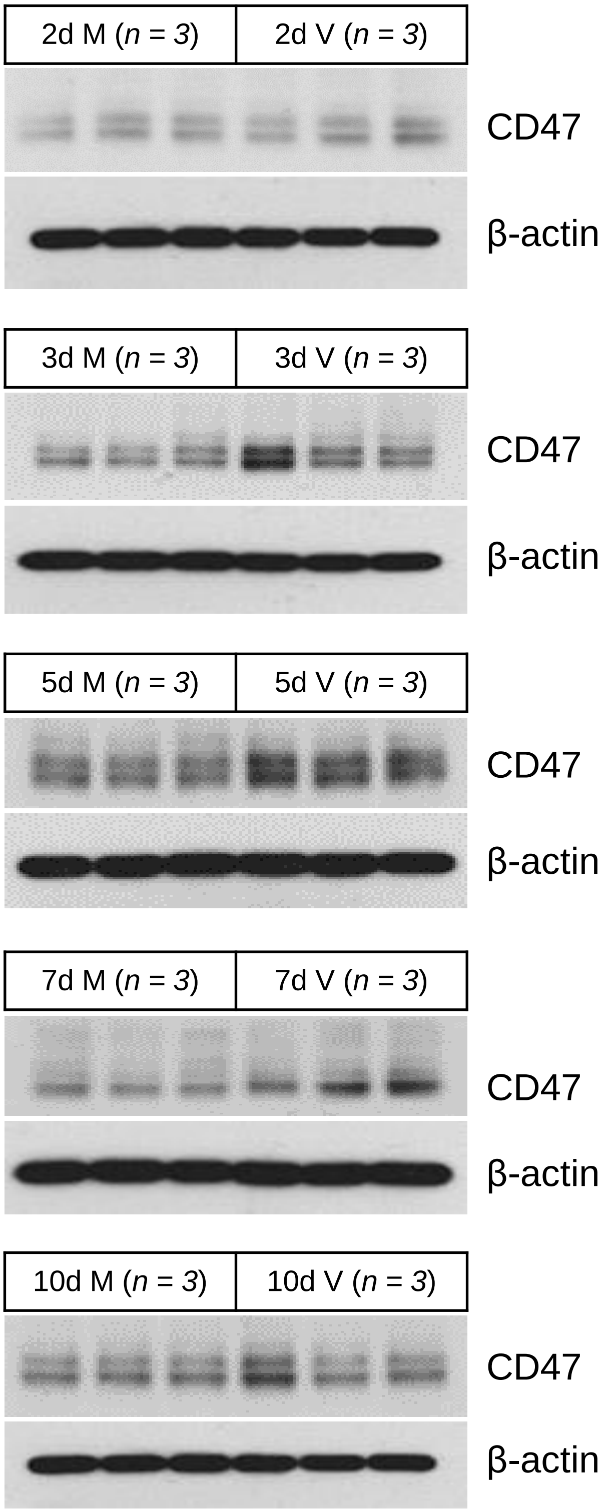

2 set post IAV infection day lung sample western blot data (mock, *n = 15* / IAV infected, *n = 15*)

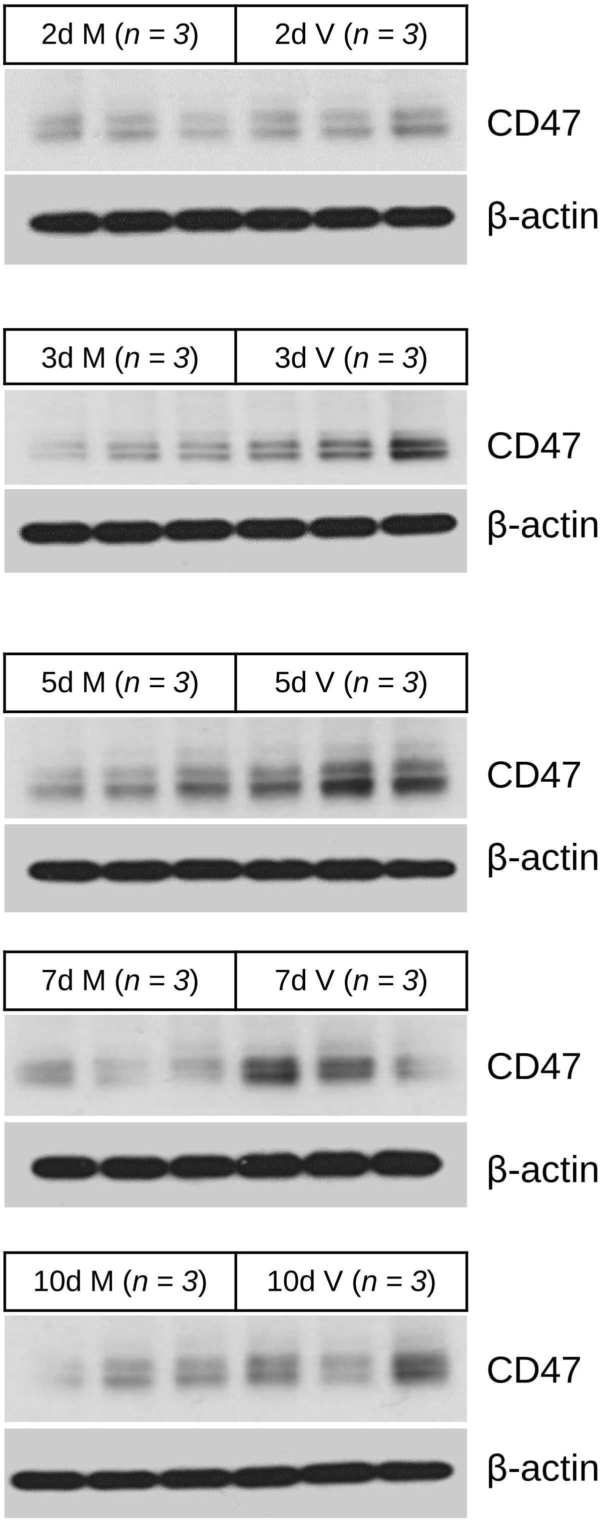

Fig. S8f

Western blot bands (in vivo)

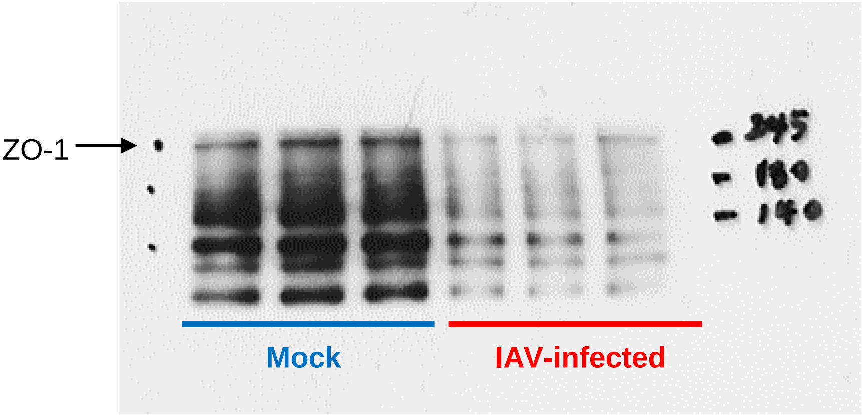

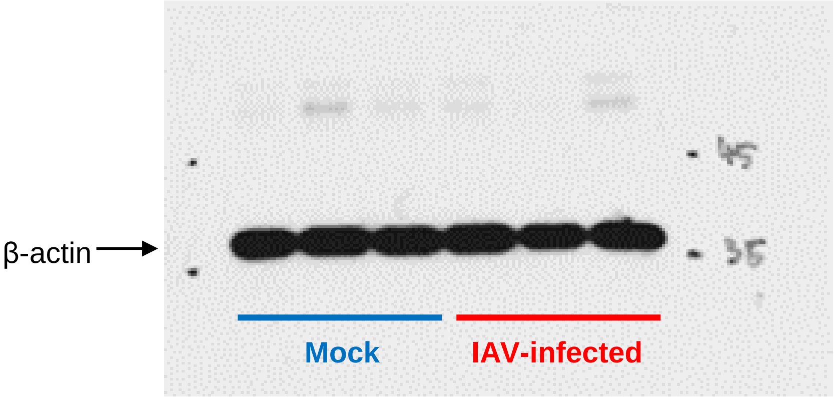

Fig. S8f

DAB staining

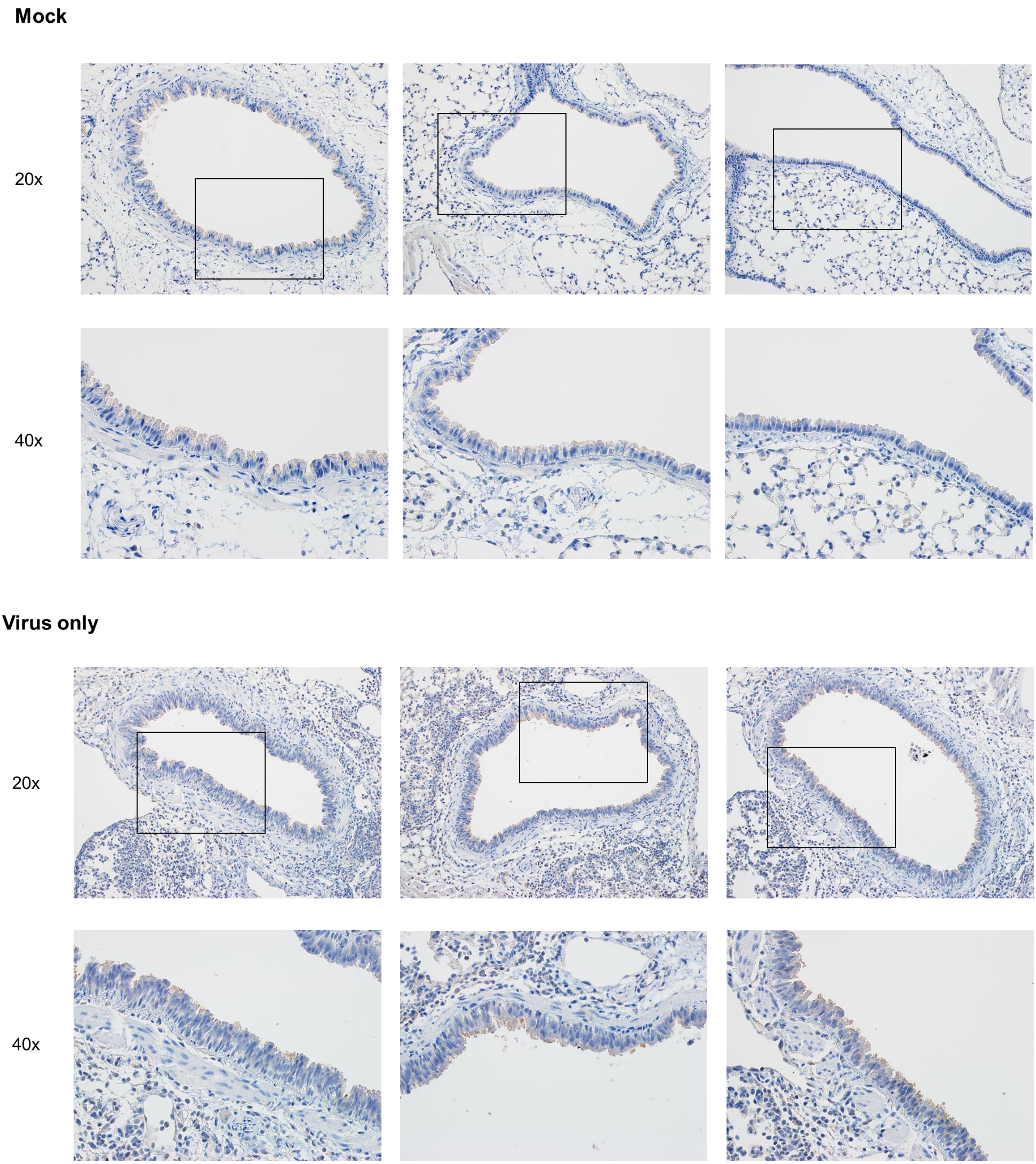

Fig. S8g

Western blot bands (HBECs)

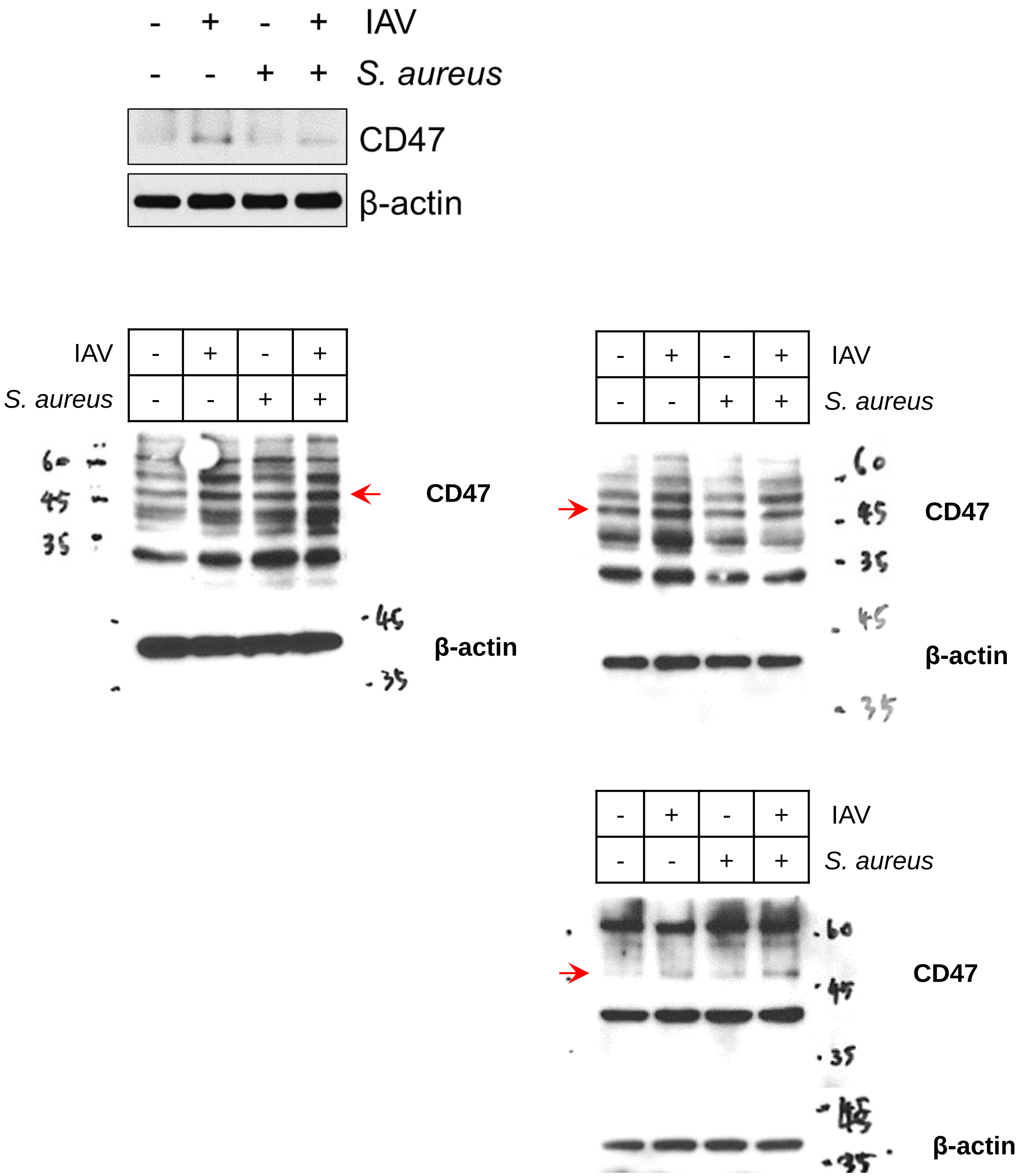

Fig. S8g (continued)

Western blot bands (HBECs)

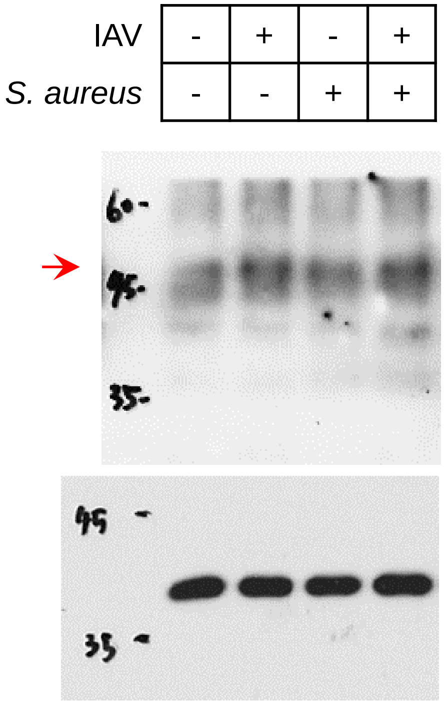

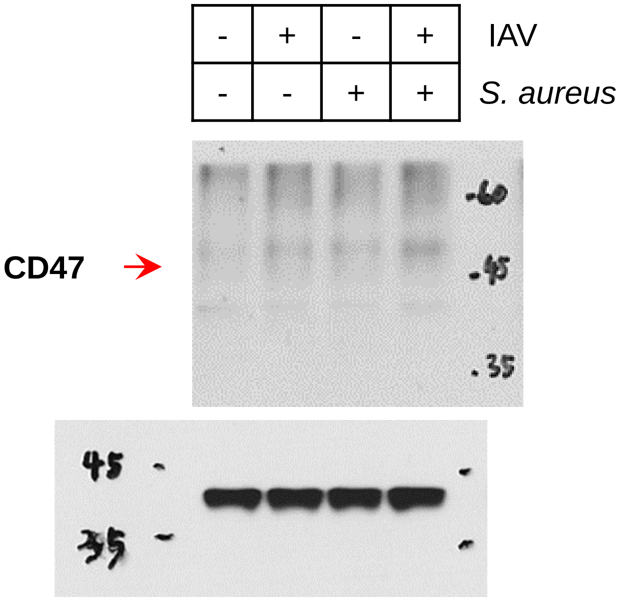

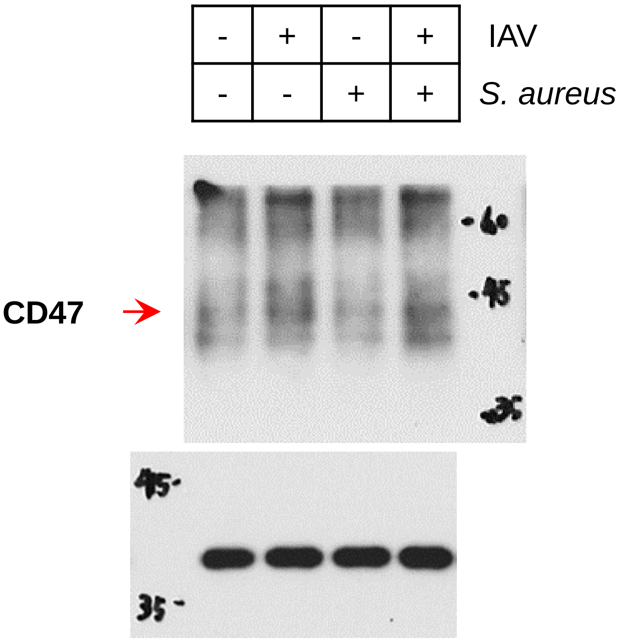

Fig. S8e

H&E staining

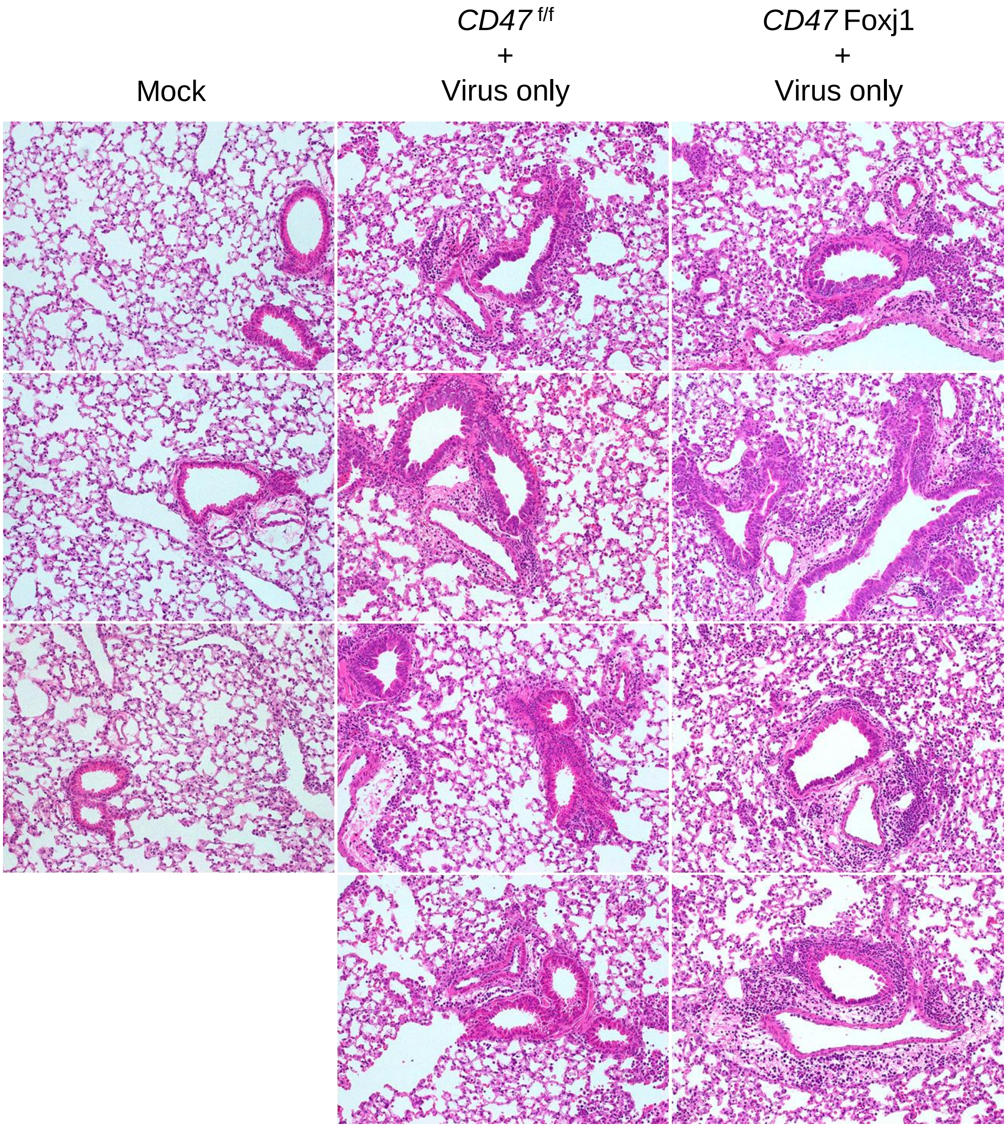

Fig. S10d

H&E staining

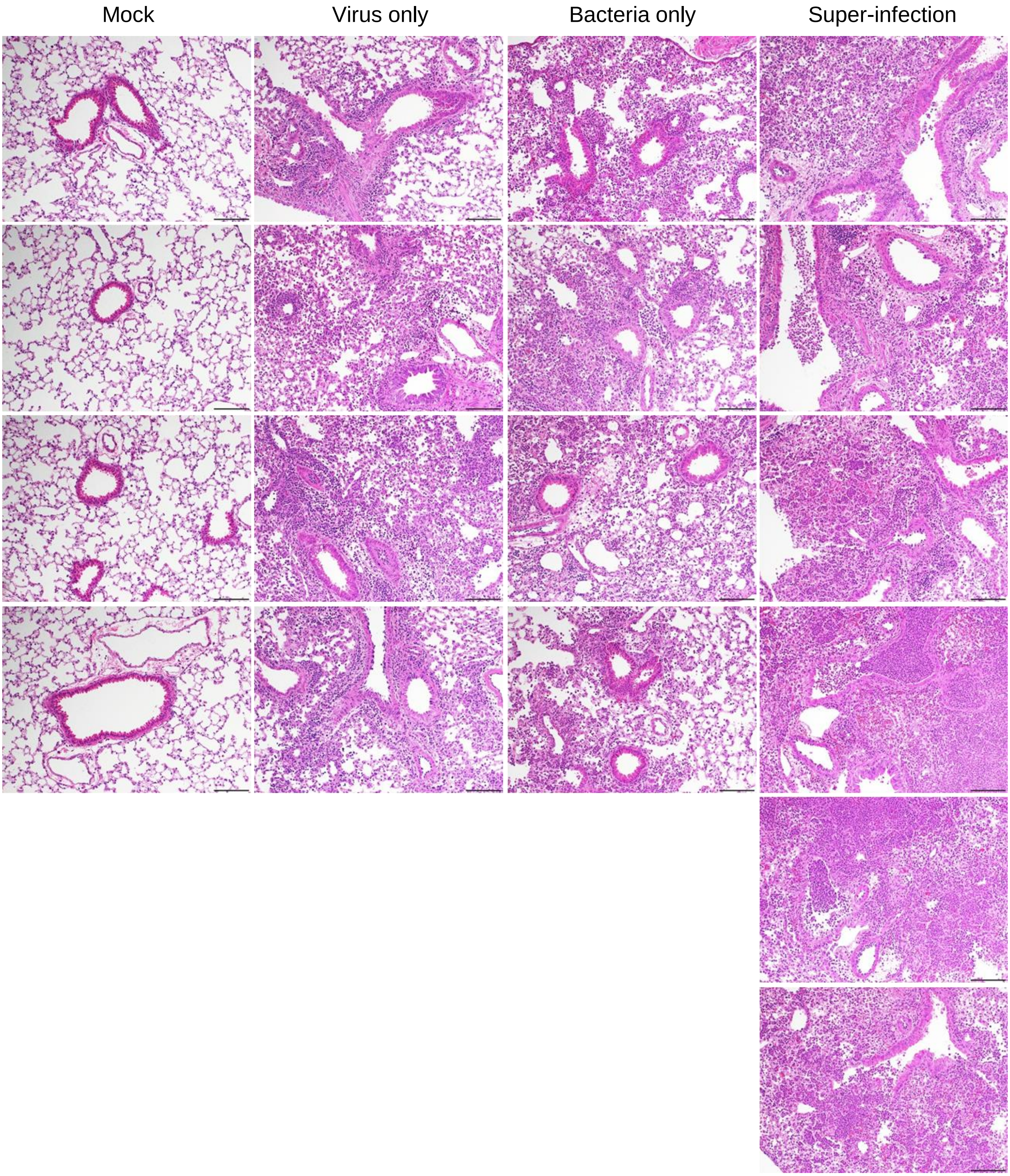

Supplement: Supplementary file 4 — Source Data [file 41467_2024_47963_MOESM4_ESM.zip › Source Data_NCOMMS-23-04701C.pdf]
